# Turning a hot spot into a cold spot: polarization-controlled Fano-shaped local-field responses probed by a quantum dot

**DOI:** 10.1038/s41377-020-00398-1

**Published:** 2020-09-21

**Authors:** Juan Xia, Jianwei Tang, Fanglin Bao, Yongcheng Sun, Maodong Fang, Guanjun Cao, Julian Evans, Sailing He

**Affiliations:** 1 Centre for Optical and Electromagnetic Research, State Key Laboratory of Modern Optical Instrumentation, National Engineering Research Center for Optical Instrumentation, JORCEP, College of Optical Science and Engineering, Zhejiang University, 310058 Hangzhou, China; 2 School of Physics, Huazhong University of Science and Technology, 430074 Wuhan, China; 3 Centre for Optical and Electromagnetic Research, ZJU-SCNU Joint Center of Photonics, South China Academy of Advanced Optoelectronics, South China Normal University, 510006 Guangzhou, China; 4 Department of Electromagnetic Engineering, School of Electrical Engineering, Royal Institute of Technology, S-100 44 Stockholm, Sweden

**Keywords:** Quantum optics, Nanophotonics and plasmonics, Sub-wavelength optics

## Abstract

Optical nanoantennas can convert propagating light to local fields. The local-field responses can be engineered to exhibit nontrivial features in spatial, spectral and temporal domains, where local-field interferences play a key role. Here, we design nearly fully controllable local-field interferences in the nanogap of a nanoantenna, and experimentally demonstrate that in the nanogap, the spectral dispersion of the local-field response can exhibit tuneable Fano lineshapes with nearly vanishing Fano dips. A single quantum dot is precisely positioned in the nanogap to probe the spectral dispersions of the local-field responses. By controlling the excitation polarization, the asymmetry parameter *q* of the probed Fano lineshapes can be tuned from negative to positive values, and correspondingly, the Fano dips can be tuned across a broad spectral range. Notably, at the Fano dips, the local-field intensity is strongly suppressed by up to ~50-fold, implying that the hot spot in the nanogap can be turned into a cold spot. The results may inspire diverse designs of local-field responses with novel spatial distributions, spectral dispersions and temporal dynamics, and expand the available toolbox for nanoscopy, spectroscopy, nano-optical quantum control and nanolithography.

## Nanoantennas: near-field light spectrum controlled and probed

The sharp tip of an atomic force microscope (AFM) has helped researchers develop a new technique for manipulating light-sensitive nanoantennas. Recent studies have shown that engineering tiny gaps between surface-bound gold nanorods is an effective way to focus optical radiation and convert it into locally concentrated energy. Researchers in China have now used an AFM tip to position a semiconductor quantum dot into the gap between two gold nanorods, and controlled and read out the spectrum of the localized energy. When the nanoantenna is exposed to a linearly polarized light, the gap hosts a hot spot with a Lorentz-type spectrum, and the quantum dot is excited. An elliptically polarized light, on the other hand, turns the hot spot into a cold spot with a Fano-type spectrum, and the quantum dot is shielded from light exposure.

## Introduction

Optical nanoantennas^1^ can interact strongly with light to efficiently convert propagating light to local fields. The local-field responses can have nontrivial features in spatial, spectral and temporal domains. Spatially, the local-field responses of nanoantennas usually feature subwavelength hot spots, which have been extensively applied to enhance light–matter interactions^2–10^ and provide high spatial resolutions^10–19^ for nano-optical applications. Complementary to local-field hot spots, the concept of local-field cold spots has also been proposed^20–23^. Cold spots with strongly suppressed local fields could serve as building blocks for highly inhomogeneous near-field distributions, which would be highly desirable for nano-optical quantum control^10,11,24^. Spectrally and temporally, the local-field responses of nanoantennas feature broad bandwidths and fast dynamics. In the local-field responses, coherence is sustained and the spectral and temporal properties are related by Fourier’s principle. By engineering the local-field responses in the spatial, spectral and temporal domains, researchers have been exploiting these nontrivial features of nanoantennas to bring spectroscopy and ultrafast physics to the nanoscale^11,25–29^.

Local-field interferences play a key role in the engineering of the local-field responses. By controlling the local-field interferences, researchers have demonstrated local-field responses with various spatial distributions, spectral dispersions and temporal dynamics^10,17,26,27,30–34^. Different degrees of freedom of the excitation light have been used to control the local-field interferences, such as the polarization^10^^,^^26^^,^^30^, the beam shape and beam position^27,31,32^, and the incidence direction^17,33,34^. Despite the remarkable progress, achieving fully controllable local-field interferences remains a major challenge. A fully controllable local-field interference should be controllable between a constructive interference and a complete destructive interference. This would bring unprecedented benefit for the engineering of the local-field responses. A local-field hot spot could be turned into a cold spot with strong local-field suppression. The dynamic range or contrast ratio could be remarkably enhanced. Intriguingly, if the local-field interference between a resonant response and a flat response could be made fully controllable, then the spectral dispersion of the local-field response could be controlled to exhibit tuneable Fano lineshapes^35–39^ with vanishing Fano dips.

For experimental study of the local-field responses, it is crucial to probe the local fields at specified spatial and spectral positions. Nonlinear signals from metals, such as two-photon photoemission electrons^26^, two-photon photoluminescence^27^ and second harmonic signals^40^, can be used for spatial and spectral local-field probing. However, since these probing signals are from a metal, they can only probe the local-field response inside the metal. The local-field response outside a metal is usually of interest when matter other than the metal itself is involved in antenna-controlled light–matter interactions. To probe the local field outside a metal, an external probe is needed. Quantum emitters can be used as tiny sensors to probe local-field intensities^10,41–45^. When a quantum emitter is placed in a local field, it is excited by the local field, and its photoluminescence intensity can reveal the local-field response through comparison with its photoluminescence intensity excited directly by the incident light. To unambiguously probe the local-field response at a specified local site of interest, the emitter must be deterministically positioned with acceptable precision. To probe the spectral dispersions of the local-field responses, the emitter should have a broadband absorption spectrum. To be quantitative, relevant parameters of the emitter, such as its quantum yield, fluorescence blinking behaviour and transition dipole orientation, must be appropriately taken into account. Importantly, the emitter as a field probe should be protected from being significantly influenced by factors that are difficult to quantify, such as fluorescence quenching by defects.

Here, we design nearly fully controllable local-field interferences in the nanogap of a nanoantenna, and experimentally demonstrate that in the nanogap, the spectral dispersion of the local-field response can exhibit tuneable Fano lineshapes with nearly vanishing Fano dips, implying that the hot spot in the nanogap can be turned into a cold spot. The nanoantenna is an asymmetric dimer of colloidal gold nanorods (GNRs), with a nanogap between the GNRs. The local-field response in the nanogap has the following features: first, local field can be excited by both orthogonal polarizations; second, the local-field polarization has a negligible dependence on the excitation polarization; third, the local-field response is resonant for one excitation polarization, but nonresonant for the orthogonal excitation polarization. The first two features make the local-field interferences nearly fully controllable. The third feature further enables Fano-shaped local-field responses. A single quantum dot (QD) is encapsulated in a silica shell and deterministically positioned in the nanogap, which meets all the listed requirements for quantitatively probing the spectral dispersion of the local-field response at a specified local site.

## Results

### Mechanism for local-field interferences and Fano-shaped local-field responses

To make the local-field interferences nearly fully controllable, the nanoantenna should have some local region where the local field can be excited by both orthogonal polarizations, and at the same time, the local-field polarization should have a negligible dependence on the excitation polarization. Let us consider a nanoantenna on a substrate in the *x*–*y* plane, and consider normally incident plane-wave excitation light **E**_exc_ with an arbitrary elliptical polarization and a unity amplitude, which can be expressed as a coherent superposition of its *x*-polarization component and *y*-polarization component

1 $${\mathbf{E}}_{\mathrm {exc}} = \cos\theta \,{\mathbf{e}}_x + {\mathrm {e}}^{{\mathrm{i}}\varphi }\sin\theta \,{\mathbf{e}}_{\it{y}}$$

where **e**_*x*_ and **e**_*y*_ are the unit vectors in the *x* and *y* directions, and tan*θ* and *φ* define the amplitude ratio and phase difference between the *y*- and *x*-polarization components of the excitation light. Under the excitation by **E**_exc_, the local field can be expressed as

2 $${\mathbf{E}} = \cos\theta \,{\mathbf{E}}^ \leftrightarrow + {\mathrm{e}}^{{\mathrm{i}}\varphi }\sin\theta \,{\mathbf{E}}^ \updownarrow$$

which is the coherent superposition of the local fields excited by the *x*- and *y*-polarization components of **E**_exc_. Here **E**^↔^ (**E**^↕^) denotes the local field under the excitation by *x*-polarized (*y*-polarized) light with a unity amplitude. It should be noted that **E**^↔^, **E**^↕^ and **E** include not only the scattered field but also the incident field. In this work, when we discuss the local field or local-field response, we refer to the *total* local field and not only to the scattered local field. If the nanoantenna has some local region where **E**^↔^ and **E**^↕^ have similar local-field polarizations (denoted ${\hat{\mathbf u}}$), then the local field excited by the *x*-polarization component of **E**_exc_ and that excited by the *y*-polarization component of **E**_exc_ can effectively interfere in this region, and the interference can be controlled by the excitation polarization. Nearly complete destructive local-field interference (i.e., nearly complete local-field suppression) can be achieved in this region when the excitation polarization satisfies the conditions

3 $$\theta = \arctan\left| {{\mathbf{E}}^ \leftrightarrow } \right|/\left| {{\mathbf{E}}^ \updownarrow } \right|,\,\varphi = \phi ^ \leftrightarrow - \phi ^ \updownarrow + 180^ \circ$$

where |**E**^↔^| (|**E**^↕^|) is the magnitude of **E**^↔^ (**E**^↕^), and *ϕ*^↔^ (*ϕ*^↕^) is the phase of the component of **E**^↔^ (**E**^↕^) along the major axis of its polarization ellipse. Equation (3) gives the excitation polarization for optimal local-field suppression. The orthogonal polarization (*θ*′, *φ*′) = (90° − *θ*, *φ* − 180°) leads to optimal local-field enhancement.

Now we have elucidated the conditions for achieving nearly fully controllable local-field interferences, but without consideration of the spectral dispersion. To make the spectral dispersion exhibit a Fano lineshape, the local-field response should be resonant for one excitation polarization (here, we assume *y*-polarization) but nonresonant for the orthogonal excitation polarization (here we assume *x*-polarization). Then, the local-field response under *y*-polarized excitation can be approximately described by a Lorentz resonance ${\mathbf{E}}^ \updownarrow = Ae^{{\mathrm{i}}\delta _{\mathrm{A}}}\omega _0/\left( {\omega - \omega _0 + {\mathrm{i}}\gamma {\mathrm{/}}2} \right){\hat{\mathbf u}}$, while the local-field response under *x*-polarized excitation can be approximately described by a flat response ${\mathbf{E}}^ \leftrightarrow = B{\mathrm e}^{{\mathrm{i}}\delta _{\mathrm{B}}}{\hat{\mathbf u}}$. Here, *A*, *B*, *δ*_A_ and *δ*_B_ are real-valued constants in the spectral range of interest, and *ω*_0_ and *γ* are the resonant frequency and linewidth of the Lorentz resonance, respectively. The resonant response **E**^↕^ and flat response **E**^↔^ interfere with each other in the near field according to Eq. (2), with their amplitude ratio and phase difference controlled by the excitation polarization. This is the near-field analogy of the Fano resonance, where resonant scattering interferes with flat scattering to produce an asymmetric spectral lineshape^35–38^. Indeed, if complete destructive interference (or local-field suppression) is achieved at an arbitrarily specified frequency *ω*_s_ using the excitation polarization given by Eq. (3), then the spectral dispersion of the local-field response is simply the well-known Fano lineshape (see Supplementary Section S1 for the derivation)

4 $$\left| {\mathbf{E}} \right|^2 \propto \frac{{\left( {{\it{\Omega }} + q} \right)^2}}{{{\it{\Omega }}^2 + 1}}$$

where Ω = 2(*ω* − *ω*_0_)/*γ* is the dimensionless frequency and *q* = 2(*ω*_0_ − *ω*_s_)/*γ* is the Fano asymmetry parameter. A Fano resonance features high-dynamic-range dispersion, quickly changing from an enhanced response (due to constructive interference) to a suppressed response (due to destructive interference) in the spectral domain. The spectral position of the Fano dip is determined by *q* via Ω = −*q*. The relation between *q* and *ω*_s_ indicates that *q* can be dynamically tuned by simply controlling the excitation polarization.

### Antenna design for Fano-shaped local-field responses

**Fig. 1:**
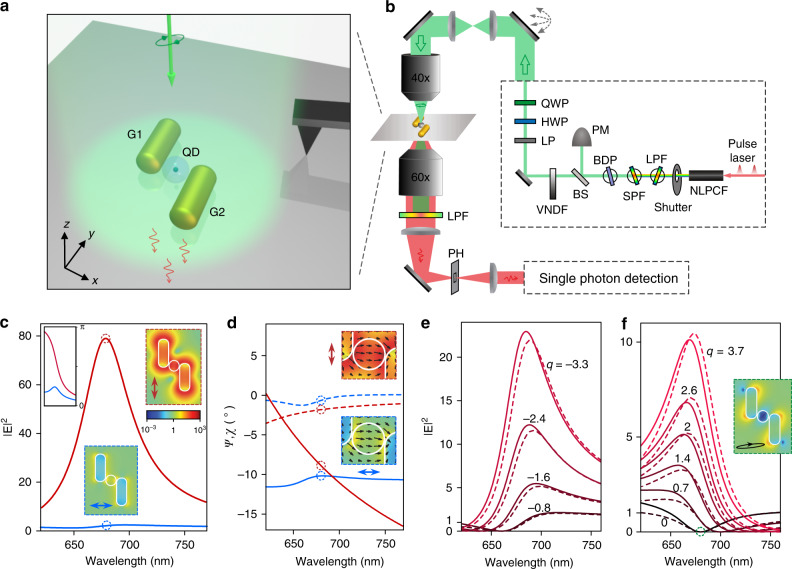
QD-loaded nanoantenna for polarization-controlled Fano-shaped local-field responses. **a** Schematics of the QD-loaded nanoantenna excited by a light beam with controlled polarization. **b** Experimental setup (Supplementary Section S2). **c** Simulated spectral dispersions of the local-field intensity responses and phase responses (upper-left inset) in the nanogap under *x*-polarized (blue curve) and *y*-polarized (red curve) excitation. The upper-right inset and lower inset show the electric field intensity distributions at 680nm under *y*- and *x*-polarized excitation. **d** Simulated spectral dispersions of the local-field polarization parameters *ψ* (solid curves) and *χ* (dashed curves) under *x*-polarized (blue curves) and *y*-polarized (red curves) excitation. The upper and lower insets show the electric field distributions at 680nm under *y*- and *x*-polarized excitation. **e**, **f** Simulated spectral dispersions of the local-field intensity responses under elliptically polarized excitations that are obtained to achieve local-field suppression at specified wavelengths. The dashed curves are the fits by Fano lineshapes, with the Fano asymmetry parameter *q* given beside the curves. The inset shows the field intensity distribution for the Fano dip at 680nm (marked by a green dashed circle), with the excitation polarization shown in the lower-left corner. All the field distributions share the same colormap shown in panel **c**

According to the design rules elucidated above, we propose a QD-loaded nanoantenna as a proof-of-principle design, as shown in Fig. 1a. The nanoantenna consists of two chemically synthesized colloidal GNRs, G1 and G2. The two GNRs form an asymmetric dimer with a nanogap. A laser beam with controlled polarization excites the antenna modes. A single colloidal QD is placed in the nanogap to spectrally probe the local-field response. The QD-loaded nanoantenna can be assembled using the nanomanipulation technique^10^. Relevant optical characterizations can be performed with the experimental setup shown in Fig. 1b, including polarization-controlled and wavelength-controlled excitation and single QD fluorescence detection (see Supplementary Section S2 for details of the experimental setup).

The antenna is designed to allow polarization-controlled effective interference between a resonant local-field response and a flat local-field response in the nanogap, which then enables tuneable Fano-shaped local-field responses. On the one hand, the local-field response is resonant for *y*-polarized excitation but roughly flat for *x*-polarized excitation, as indicated by the numerically simulated local-field spectral responses in Fig. 1c. Under *y*-polarized excitation, the intensity response (red curve) shows a strong enhancement peak, and the phase response (red curve in the inset) undergoes an abrupt change by ~π across the resonance peak, while under *x*-polarized excitation, both the intensity response (blue curve) and phase response (blue curve in the inset) are roughly flat. As shown by the local-field intensity distributions at the resonant wavelength of ~680 nm, under *y*-polarized excitation, the nanogap hosts a hot spot with strong local-field enhancement (upper-right inset), while under *x*-polarized excitation, the local-field enhancement is moderate (lower inset). On the other hand, the local-field polarization in the nanogap is nearly independent of the excitation polarization, as indicated by the numerically simulated local-field polarizations in Fig. 1d. In the *x–y* plane at the same height as the centre of the QD, the *z*-components of the local-field responses are negligible (Supplementary Fig. S1), i.e., **E**^↔^ and **E**^↕^ are polarized in the *x–y* plane. Thus the local-field polarizations can be described by a polarization ellipse in the *x–y* plane with ellipse parameters *ψ* and *χ*, where *ψ* is the orientation angle of the polarization ellipse defined as the angle between the major axis of the polarization ellipse and the *x*-axis, and *χ* is the ellipticity angle of the polarization ellipse defined as the arc tangent of the ratio of the ellipse’s minor axis to major axis, whose sign defines the rotation direction. The curves of *χ* are close to 0°, which indicates that **E**^↔^ and **E**^↕^ are both nearly linearly polarized. The curves of *ψ* show that the local-field polarization angles of **E**^↔^ and **E**^↕^ are similar in the spectral range of interest. Around the resonant wavelength, the local-field polarizations of **E**^↔^ and **E**^↕^ are nearly identical, as is also indicated by the instantaneous electric field vectors plotted in the insets of Fig. 1d.

The resonant response **E**^↕^ and flat response **E**^↔^ can then effectively interfere in the near field according to Eq. (2), with their amplitude ratio and phase difference controlled by the excitation polarization. With the numerically simulated dispersions of the amplitude and phase of **E**^↔^ and **E**^↕^, we can readily calculate according to Eq. (3) the required excitation polarization for achieving optimal destructive local-field interference (or local-field suppression) at an arbitrarily specified excitation wavelength. The required excitation polarization parameters and the corresponding local-field suppression factors, as functions of the specified excitation wavelength, can be found in Supplementary Section S4. Figure 1e, f shows the local-field spectral responses for a series of excitation polarizations. They can indeed be fitted by Fano lineshapes (dashed curves with the *q* values given; see Supplementary Section S1 for the fitting method), with Fano dips at the specified wavelengths (see Supplementary Fig. S3 with the zoomed-in vertical axis) and Fano asymmetry parameters tuneable from negative to positive values. Note that at the Fano dips the local field is nearly vanishing, which means that the backgrounds of the Fano lineshapes are very low. The low background and high tunability of the Fano lineshapes indicate that local-field interference can be designed to be nearly fully controllable. The residual local-field responses at the Fano dips are due to the slight misalignments between **E**^↔^ and **E**^↕^ (Supplementary Section S5). Local-field distributions (cf. the inset of Fig. 1f) show that at the Fano dips the nanogap hosts a deep-subwavelength cold spot with strongly suppressed local field. This indicates that by changing the excitation polarization, the hot spot with strong local-field enhancement (cf. Fig. 1c) can be turned into a cold spot with strong local-field suppression (cf. Fig. 1f).

If the local field is excited by a broadband ultrafast pulse, then the spectrum of the local field can be tuned by controlling the local-field spectral response, as demonstrated above. Since coherence is sustained in the local-field response^25–27^, and the spectral and temporal properties are related by Fourier’s principle, the temporal dynamics of the local field can then be accordingly tuned by simply controlling the excitation polarization. The polarization-controlled spectral distributions and temporal evolutions of the ultrafast local field are given in Supplementary Section S6.

### Fabrication of the QD-loaded nanoantenna

**Fig. 2:**
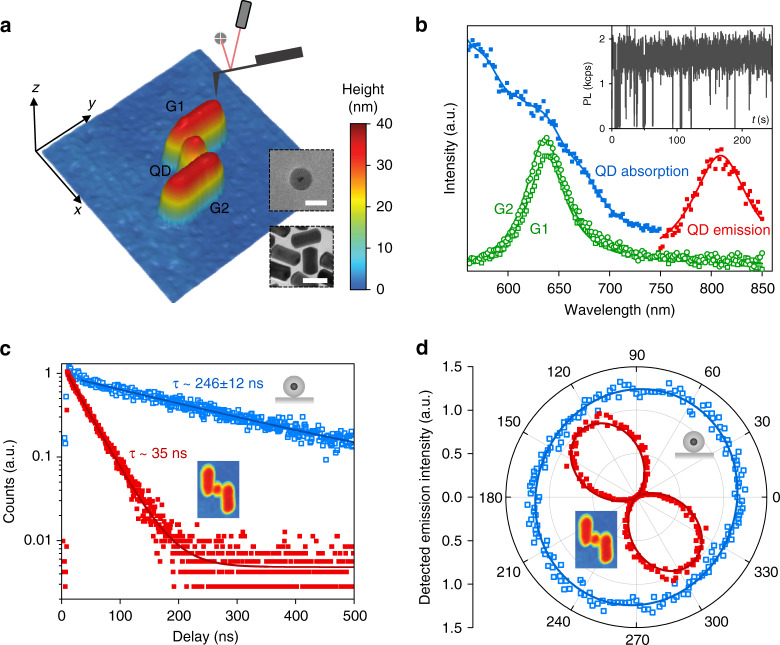
Fabrication and fluorescence characterization of the QD-loaded nanoantenna. **a** AFM image of the fabricated QD-loaded nanoantenna. Upper inset: TEM image of GNRs (scale bar, 50nm); Lower inset: TEM image of a silica-encapsulated QD (scale bar, 30nm). **b** Optical characterization of the constituent GNRs and QD. Measured dark-field scattering spectra of GNRs G1 (green squares) and G2 (green circles) (solid curves are simulated spectra). Measured absorption spectrum (blue; solid curve is the smoothed curve) and emission spectrum (red, measured when excited at 670nm; solid curve is a Lorentz fit) of the QD. The upper-right inset shows a time trajectory of the fluorescence from the QD. **c** Fluorescence lifetime of the QD before (blue) and after (red) coupling with the nanoantenna. The solid curves are monoexponential decay fittings. **d** Polar plots of the emission polarization measurements (detected intensity versus angle of the linear polarizer) of the QD before (blue) and after (red) coupling with the nanoantenna. The blue continuous curve is a sinusoidal fit. The red continuous curve is the simulated result

The designed QD-loaded nanoantenna is deterministically fabricated based on the nanomanipulation technique (see ‘Methods' for the fabrication process). Figure 2a shows an atomic force microscope (AFM) image of the fabricated sample. The two GNRs (Nanopartz Inc.) have similar diameters (~32 nm for G1; ~34 nm for G2) and similar lengths (~78 nm for G1; ~80 nm for G2). The GNRs have similar plasmonic resonance peaks around the wavelength of 638 nm, as indicated by the measured dark-field scattering spectra shown in Fig. 2b. The QD is a colloidal CdSeTe/ZnS core–shell QD (Invitrogen, Qdot 800 ITK carboxyl) encapsulated in a silica shell with a thickness of ~10 nm^46^. The total diameter of the silica-encapsulated QD is ~31 nm. The width of the nanogap between G1 and G2 is ~36 nm. Due to fabrication imperfections, the position of the QD deviates slightly from the centre of the nanogap by ~2 nm towards G1. The method for estimating the structural parameters of the fabricated sample can be found in Supplementary Section S7. Clear binary fluorescence intermittency can be identified in the fluorescence time trajectory of the QD (inset of Fig. 2b), with its ‘off’-state fluorescence signal down to the background signal level, which is characteristic of a single QD^47^. The silica shell protects the QD from being quenched by surface defects, which makes the QD quantitatively reliable as a local-field probe. The intrinsic ‘on’-state quantum yield of the QD on the substrate can be assumed to be unity (Supplementary Section S9). The QD has a broadband and smooth absorption spectrum (Fig. 2b), which makes it suitable for spectral probing. The absorption spectrum is measured through photoluminescence excitation (PLE) spectroscopy^48,49^. The emission spectrum of the QD exhibits a peak at ~808 nm (Fig. 2b), which has no noticeable dependence on the excitation wavelength in the spectral range of interest (560–750 nm).

### Fluorescence tailoring by the nanoantenna

Since local-field probing relies on the detection of the fluorescence of the QD, it is important to know the fluorescence properties, which may be significantly influenced by the nanoantenna due to the Purcell effect^10,50–52^. Antenna modes can provide a large local density of photonic states (LDOS), and then, according to Fermi’s golden rule, the radiative decay rate of the QD will be enhanced via energy transfer to the antenna modes^53,54^. Owing to the broad bandwidth of the antenna resonance, although the emission wavelength of the QD (~808 nm) is far detuned from the resonant peak of the nanoantenna (~645 nm), the measured fluorescence lifetime of the QD is still shortened by ~7-fold (Fig. 2c), which can be explained by the simulated Purcell factor of ~8.5 (Supplementary Section S10). With such a significant Purcell effect, the fluorescence of the QD should predominantly transfer to the antenna modes and the far-field radiation is expected to be tailored by the antenna. Far-field polarization analysis (Fig. 2d) shows that the collected photons have a high degree of linear polarization of ~0.97, in stark contrast to the unpolarized photon emission before the QD is coupled to the nanoantenna. The polarization angle agrees with the simulation. This indicates that the fluorescence indeed predominantly transfers to the antenna modes and the far-field radiation properties are governed by the antenna. Note that here, the shortened lifetime and tailored far-field radiation polarization are both well explained by the Purcell effect; nonradiative energy transfer to surface recombination centres or carrier tunnelling to metal is unlikely to play a significant role in our experiment owing to the thick silica shell of the QD (see Supplementary Section S11 for a detailed analysis).

### Local-field spectral probing using the QD

Through PLE spectroscopy, i.e., measuring the photoluminescence intensity as a function of excitation wavelength, the absorption spectrum can be obtained as *σ*(*λ*) = *I*_em_(*λ*)/[*ρ*_ex_(*λ*)*ηξ*], where *ρ*_ex_(*λ*) is the intensity of the excitation light (photons s^−1^ cm^−2^) as a function of excitation wavelength, *I*_em_(*λ*) is the detected photon count rate (photons s^−1^) as a function of excitation wavelength, *η* is the ‘on’-state quantum yield and *ξ* is the fluorescence detection efficiency^48^. Before the QD is coupled to the antenna, *η* is taken as unity (Supplementary Section S9) and *ξ* is determined to be ~0.97% (Supplementary Section S8). After the QD is coupled to the antenna, its fluorescence is tailored by the antenna, and therefore *η* and *ξ* may change. According to numerical simulations, *η* is reduced to ~56% due to the finite radiation efficiencies of the antenna modes (Supplementary Section S10). Although the effective radiation dipole is governed by the antenna modes, *ξ* will not change because the effective radiation dipoles of the antenna modes are still in the horizontal plane. The experimental details for measurement of absorption spectra can be found in the ‘Methods’.

By measuring the absorption spectra of the QD before and after it couples to the antenna, the substrate-normalized local-field spectral response of the nanoantenna can be obtained as the ratio *σ*_ant_(*λ*)/*σ*_sub_(*λ*), where *σ*_ant_(*λ*) denotes the absorption spectrum after the QD has been coupled to the antenna and *σ*_sub_(*λ*) denotes the absorption spectrum when the QD is on the substrate before being coupled to the antenna. The experimentally probed substrate-normalized local-field response corresponds to the quantity |**E**_ant_|^2^/|**E**_sub_|^2^, where **E**_ant_ and **E**_sub_ are the incidence-normalized local field at the antenna and at the substrate surface, respectively. The local-field response at the substrate surface is featureless, so it can be reliably theoretically or numerically estimated. Then, the incidence-normalized local-field response can be calculated from the substrate-normalized local-field response. For experimental studies, substrate normalization is usually used, so in our experimental demonstrations, we still use the substrate-normalized local-field response, which is also denoted as excitation enhancement factor. An excitation enhancement factor less than one means that the local field is suppressed, and its reciprocal is denoted as the excitation suppression factor.

For local-field probing, the QD is excited far from its band edge, and here, its bright plane is made nearly horizontal, so the excitation of the QD is nearly polarization independent in the *x–y* plane^55^. Thus, the QD can probe the intensity of the local field in the *x*–*y* plane in an isotropic manner. In our study, regardless of whether the QD is excited directly by the incident light or has been coupled to the antenna, the local field at the QD is predominantly in the *x*–*y* plane (Supplementary Section S3), so the QD can reliably probe the local-field response. If otherwise, the *z*-component of the local field were significant, then the QD probe would somewhat overestimate the local-field intensity, because the absorption coefficient along its dark axis (which is in the *z* direction) is expected to be slightly larger than that in its bright plane due to its slightly elongated shape.

### Experimental demonstration of Fano-shaped local-field responses

**Fig. 3:**
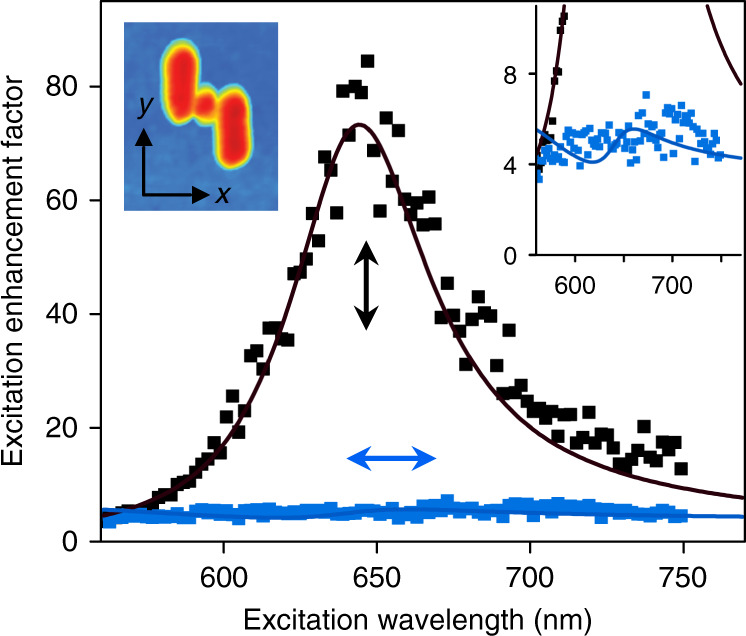
Local-field spectral responses under *x*- and *y*-polarized excitation. The blue and black data points are the measured excitation enhancement spectra (substrate-normalized local-field responses) under *x*- and *y*-polarized excitation, respectively. The solid curves are the numerically simulated spectra. The upper-left inset shows the *x*–*y* coordinates defined on the AFM image of the sample. The upper-right inset re-plots the spectra with a zoomed-in vertical axis

With the fabricated QD-loaded nanoantenna and the local-field probing technique, we are now ready to experimentally demonstrate polarization-controlled Fano-shaped local-field responses as predicted by our theoretical and numerical study. As one of the requirements for producing a Fano-shaped local-field response, a resonant local-field spectral response and a flat local-field spectral response must be experimentally shown first. To this end, we measure the excitation enhancement spectra under *y*- and *x*-polarized excitation, as shown in Fig. 3. As expected, under *y*-polarized excitation, the measured enhancement spectrum (black data points) shows a strong resonance peak at the wavelength of ~645 nm, with an excitation enhancement factor of ~80; under *x*-polarized excitation, the measured spectrum (blue data points) is rather flat, with an enhancement factor of ~5. The measured spectra agree well with the numerically simulated local-field enhancement spectra (solid black and blue curves). Note that all the numerical simulations for comparison with the experimental results use the structural parameters estimated for the fabricated sample (Supplementary Section S7).

**Fig. 4:**
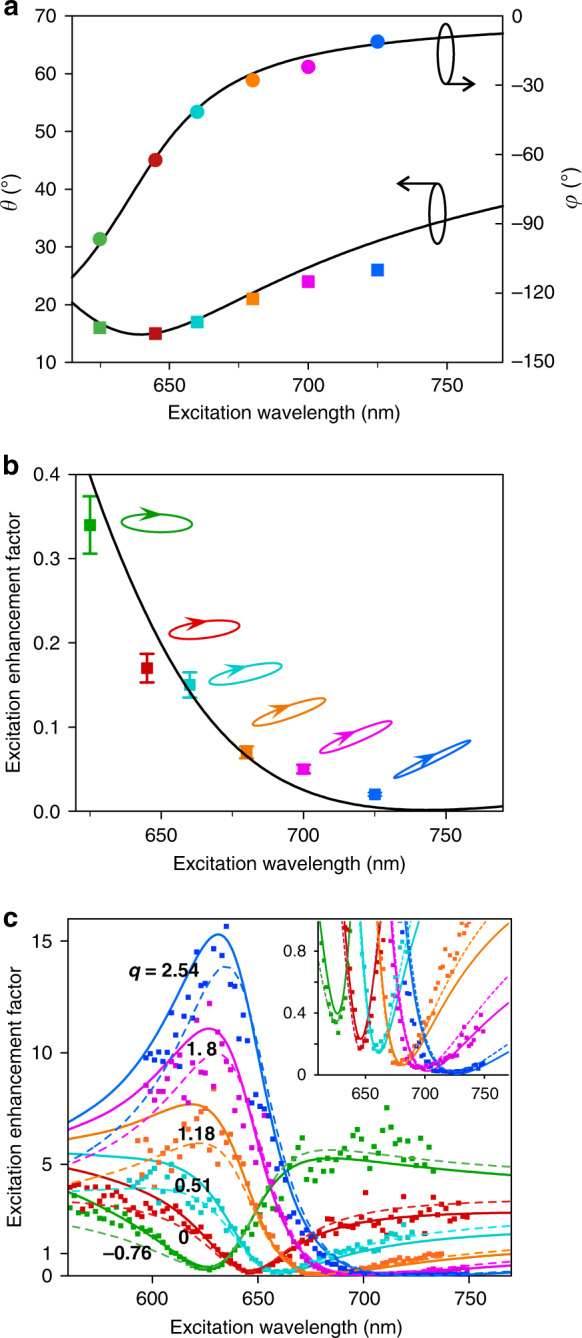
Experimental demonstration of polarization-controlled Fano-shaped local-field responses. **a** Experimentally obtained values of *θ* (coloured circles) and *φ* (coloured squares) of the excitation polarizations for achieving optimal destructive local-field interference (or local-field suppression) at specified wavelengths. The solid curves are the numerically calculated values of *θ* and *φ* according to Eq. (3). **b** Minimal excitation enhancement factors (coloured squares with error bars) achieved by using the excitation polarizations denoted by arrowed ellipses (corresponding to the experimentally obtained *θ* and *φ* in panel a). The solid black curve shows the numerically obtained minimal excitation enhancement factors. The error bars are extracted from the standard error of the time-integrated photon counting of the photoluminescence. **c** Measured excitation enhancement spectra (solid data points) for all the excitation polarizations shown in panel a. The correspondences between the spectra and the excitation polarizations are indicated by the colours. The solid curves are the numerically calculated spectra, and the dashed curves are the fits by Fano lineshapes with the *q* values given. The upper-right inset re-plots the spectra with a zoomed-in vertical axis

Then, the interference between the resonant and flat local-field responses needs to be controlled by the excitation polarization. Similar to the theoretical study, we need to find the required excitation polarizations for achieving optimal destructive local-field interference (or local-field suppression) at specified wavelengths. For any specified wavelength, we experimentally find the required excitation polarization by successively searching the elliptical polarization parameters *φ* and *θ* for a minimized photoluminescence. The experimentally obtained values of *θ* and *φ* are shown in Fig. 4a. These values agree well with the numerically calculated values. Since the numerically obtained values are calculated according to Eq. (3), this indicates that our system indeed works according to the proposed local-field interference mechanism.

The correspondingly achieved minimal enhancement factors (the reciprocals of the enhancement factors are the excitation suppression factors) are plotted as square data points in Fig. 4b. The solid black curve shows the minimal enhancement factors obtained from numerical simulations. Significant excitation suppression is experimentally achieved for all excitation wavelengths. Notably, for the excitation wavelength of 725 nm, the excitation is suppressed to 2%, i.e., an excitation suppression factor as high as 50 is experimentally achieved. The significantly higher suppression factor at 725 nm than at 645 nm is attributed to the fact that for the fabricated QD-loaded nanoantenna the local-field polarizations of **E**^↔^ and **E**^↕^ in the nanogap are more similar at 725 nm than at 645 nm (Supplementary Section S12). Although the local-field response is probed at one spatial point, numerical simulations show that a three-dimensional local-field cold spot is hosted in the nanogap (Supplementary Fig. S12).

Having experimentally obtained the required excitation polarizations, the excitation enhancement spectrum can then be measured for these excitation polarizations. The measured excitation enhancement spectra are shown in Fig. 4c. The measured enhancement spectra (square data points) agree well with the numerically calculated results (solid curves). Importantly, these experimentally measured spectra can indeed be well fitted by Fano lineshapes (dashed curves) of different *q* values depending on the excitation polarization. The Fano dips are at the specified local-field suppression wavelengths (see the inset with the zoomed-in vertical axis). Thus, we have already experimentally demonstrated that the local-field response in the nanogap can exhibit dynamically tuneable Fano lineshapes. By simply controlling the excitation polarization, the Fano asymmetry parameter *q* can be tuned from negative to positive values, and correspondingly, the Fano dip can be tuned across a broad wavelength range. Moreover, the nearly vanishing Fano dips indicate that the Fano lineshapes have low backgrounds. Fano lineshapes with a sharp change between the Fano peak (with a local-field enhancement) and the nearly vanishing Fano dip (with a local-field suppression) imply a high dynamic range in the spectral domain.

It is worth noting that if the Fano dip is far from the resonant wavelength of 645 nm (e.g., at 700, 725 nm), the response spectrum around the dip can be rather flat, which allows broadband local-field suppression. For instance, for the excitation spectrum with a dip at 725 nm, the measured excitation suppression factors all exceed 20 for excitation wavelengths spanning from 710 to 740 nm, as shown by the blue-coloured spectrum in Fig. 4c. Such broadband suppression is attributed to the relatively weak spectral dispersions of the amplitudes and phases of **E**^↔^ and **E**^↕^ at wavelengths significantly longer than the resonant wavelength of 645 nm. This behaviour is expected for a Fano-shaped response with a large value of *q*.

### Switching between enhancement and suppression

**Fig. 5:**
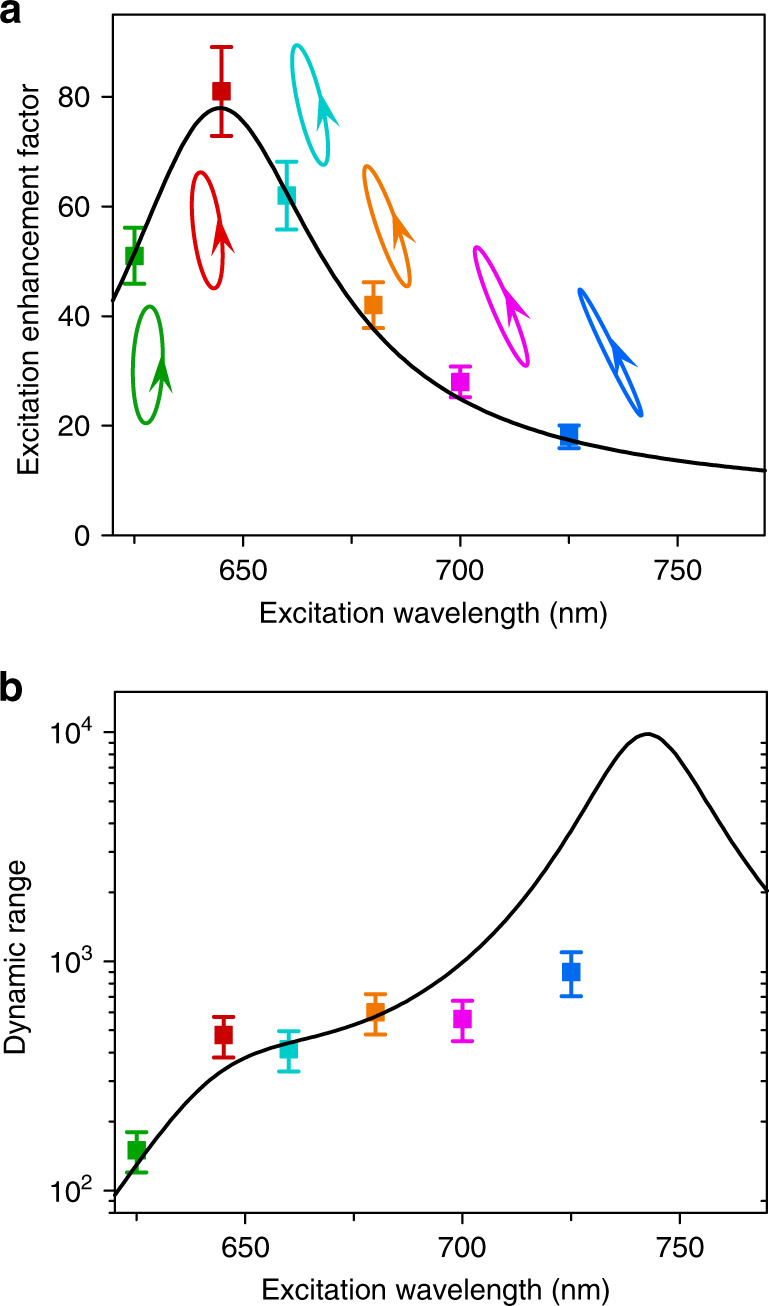
Wavelength-specified dynamic range of the local-field response. **a** Maximal excitation enhancement factors (coloured data points) and corresponding excitation polarizations (coloured polarization ellipses) for specified excitation wavelengths. The solid black curve shows the numerically obtained maximal excitation enhancement factors. The error bars are extracted from the standard error of the time-integrated photon counting of the photoluminescence. **b** Wavelength-specified dynamic range of the local-field response. The solid curve is the numerically obtained wavelength-specified dynamic range

Once the excitation polarization for optimal excitation suppression has been found, optimal excitation enhancement can then be obtained by simply using the orthogonal polarization, as shown by the polarization ellipses in Fig. 5a. The achieved maximal excitation enhancement factors are plotted as square data points in Fig. 5a. Figures 4b and 5a together indicate that the local-field response can be switched between enhancement and suppression in a broad wavelength range, implying that the hot spot in the nanogap can be turned into a cold spot. The achievable lowest excitation enhancement factor (the reciprocal of which is the achievable highest excitation suppression factor) and highest excitation enhancement factor depend on the excitation wavelength. Here, we define the wavelength-specified dynamic range of local-field response as the ratio of the highest and lowest excitation enhancement factors that can be achieved at the specified excitation wavelength. Figure 5b shows the wavelength-specified dynamic ranges for excitation wavelengths from 625 to 725 nm. At wavelengths longer than the resonant wavelength of 645 nm, although the achievable highest excitation enhancement factor is less than that achievable at the resonant wavelength (Fig. 5a), a high dynamic range can still be achieved because the achievable highest suppression factor is greater than that achievable at the resonant wavelength (Fig. 4b). For instance, at 725 nm, while the experimentally achieved highest excitation enhancement factor is ~18, the excitation suppression factor can be as high as ~50, so the dynamic range can reach ~900, which is even higher than the dynamic range at the resonant wavelength of 645 nm. The experimentally achieved wavelength-specified dynamic ranges for excitation wavelengths between 645 and 725 nm all exceed 400. Theoretically, a wavelength-specified dynamic range over 9000 can be achieved at ~740 nm, where the theoretically obtained suppression factor is as high as ~700. Experimentally, the highest suppression factor is limited to ~50 due to the limited fabrication and measurement precision, and consequently, the highest wavelength-specified dynamic range is limited to ~900. Note that the dynamic range defined above is defined at a single spatial point, different from the spatial contrast ratio. To obtain a high spatial contrast ratio, more antennas are needed. For instance, if two identical antennas, each with a high dynamic range of the local-field response, are placed together with orthogonal orientations, then we can achieve a high spatial contrast ratio, which is roughly the value of the dynamic range of each antenna (Supplementary Section S13).

## Discussion

We would like to emphasize that the demonstrated local-field control in this work is for the *total* local field, not only the *scattered* local field. The demonstrated local-field suppression means that the *total* local field is suppressed. If only the scattered local field is suppressed, then the incident field still exists, and the total field is not actually suppressed. In addition, the local-field suppression or cold spot in this work should not be confused with the dark spots that are formed due to fluorescence quenching^12,56^ or due to the highly polarization-dependent absorption of emitters^12^, which may be observed in metal-tip scanning fluorescence imaging.

We also emphasize that the local-field interference in this work should not be confused with the interference between the scattered field and the incident field, which led to weakly suppressed local fields in an earlier experimental work^44^. For interference between the scattered field and the incident field, the amplitude ratio and phase difference between them is not easily controllable. In contrast, in this work the local-field interference is between the local fields **E**^↔^ and **E**^↕^ that already include the incident field, and the amplitude ratio and phase difference between **E**^↔^ and **E**^↕^ can be fully controlled and optimized by controlling the polarization of the excitation light. Therefore, one can make the interference nearly completely destructive and thereby achieve strong local-field suppression and Fano-shaped local-field responses with nearly vanishing Fano dips. Moreover, the controllable interference also enables dynamic tuning of the local-field response, such as dynamic tuning of the Fano lineshapes and dynamic switching between suppression and enhancement.

Moreover, the local-field suppression is not related to suppressed LDOS. Actually, a suppressed local field and an enhanced LDOS are simultaneously observed in our experiment. The local-field response reveals how the incident light excites the antenna modes; therefore, it depends on the polarization of the incident light. The LDOS indicates how the emitter excites the antenna modes^53,54^; therefore, it depends on the position and orientation of the emitter but does not depend on the incident light polarization. The local-field response and LDOS are similar only when the incident light and the emitter excite the antenna modes in a similar way. However, here, since the polarization of the incident light is specially controlled, the excitation of the antenna modes by the incident light can be totally different from the excitation of the antenna modes by the emitter.

In conclusion, based on a rationally designed and deterministically assembled QD-loaded nanoantenna, polarization-controlled Fano-shaped local-field responses have been unambiguously demonstrated in a deep-subwavelength nanogap. By simply controlling the excitation polarization, the Fano asymmetry parameter *q* can be tuned from negative to positive values, and correspondingly, the Fano dip can be tuned across a broad wavelength range. Notably, at the Fano dips, the local-field intensity is strongly suppressed by up to ~50-fold, implying that the hot spot in the nanogap can be turned into a cold spot. The low background and dynamically tuneable Fano-shaped local-field responses can contribute as design elements to the toolbox for spatial, spectral and temporal local-field engineering. More importantly, the low background and high tunability of the Fano lineshapes indicate that local-field interferences can be made nearly fully controllable. Since the local-field interferences play a key role in the spatial, spectral and temporal engineering of the local-field responses^10,17,26,27,30–34^, this encouraging conclusion may further inspire diverse designs of local-field responses with novel spatial distributions, spectral dispersions and temporal dynamics, which may find application in nanoscopy^29,57^, spectroscopy^11,28^, nano-optical quantum control^10,11,24^ and nanolithography^17^. The Fano-shaped local-field response may also find application in other areas where the unique features of Fano lineshapes are desirable, such as in sensing and switching^37,38^, especially when applications can benefit from a low-background and dynamic tuning.

## Materials and methods

### AFM manipulation

An Agilent 5500 SPM system in tapping mode is used for imaging, pushing and rolling of the nanoparticles. For imaging, the scanning parameters are optimized to produce high-quality images without moving the nanoparticles. For pushing, the target vibration amplitude of the tapping tip is set to less than half the height of the nanoparticle. Then, the nanoparticle can be pushed by the AFM tip with the feedback loop turned off. For rolling, the tapping tip is pressed onto the upper part of the nanoparticle by setting its target vibration amplitude to zero. Then, the nanoparticle can be rolled by moving the AFM tip with the feedback loop turned off.

### Sample fabrication

Colloidal CdSeTe/ZnS core-shell QDs (Invitrogen, Qdot 800 ITK carboxyl) are encapsulated with silica shells through a sol–gel reaction^46^. Then colloidal GNRs (Nanopartz Inc.) and the silica-encapsulated QDs are successively transferred through spin-coating to a silica glass substrate where markers have been formed through photolithography and lift-off. Then, GNRs with a plasmonic resonance at ~640 nm are selected by measuring the dark-field scattering spectra of the GNRs (Fig. 2b). Single QDs with clear binary fluorescence intermittency (the ‘off’-state fluorescence signal is down to the background signal level, and the ‘on’-state fluorescence signal is stable) are selected by measuring the time trajectories of the fluorescence signal (inset of Fig. 2b). The selected GNRs and QDs are optically localized with respect to the markers on the substrate, which facilitates AFM localization. Then the orientation of a selected QD is manipulated by rolling the QD with the AFM tip so that the collected fluorescence is made to be nearly completely unpolarized, which means that the bright plane of the QD is nearly horizontal. After that, the QD is no longer moved. Finally, the selected GNRs are pushed with the AFM tip to assemble the nanoantenna with the QD in the nanogap (without moving the QD).

### Measurement of absorption spectra

To measure the absorption spectra *σ*_sub_(*λ*) and *σ*_ant_(*λ*) through PLE spectroscopy, a programme scans the wavelength of the excitation light. During the wavelength scan, *I*_em_(*λ*) is set at a constant level through adaptive control of *ρ*_ex_(*λ*) by adjusting a variable neutral density filter. The programme also identifies the occasional blinking of the QD by simply monitoring the sudden signal drop and waits until the QD recovers its fluorescence. The adaptive control of the excitation power guarantees a suitable excitation intensity (strong enough to guarantee an adequate signal-to-noise ratio but not strong enough to saturate the excitation or deteriorate the QD fluorescence) for every wavelength in the spectral range. This is especially important when the QD is coupled with the nanoantenna, where the local-field responses may have high-dynamic-range spectral dispersions. Note that *σ*_sub_(*λ*) and *σ*_ant_(*λ*) are obtained for the same QD: *σ*_sub_(*λ*) is measured after the QD bright plane has been manipulated to be nearly horizontal but before antenna coupling; *σ*_ant_(*λ*) is taken after antenna coupling.

### Numerical simulation

Numerical simulations are performed using a commercial finite-difference time-domain (FDTD) solver (FDTD solutions, Lumerical). The optical constants of gold used for FDTD simulations are taken from the reported experimental data^58^. Regarding the dielectric property, the silica-encapsulated QD is modelled as a homogeneous and isotropic silica sphere with a diameter of 31 nm (see Supplementary Section S14 for the influence of this approximation). A total-field scattered-field (TFSF) source is used to simulate the plane-wave excitation of the nanoantenna. Plane-wave excitation is a reasonable approximation for the Gaussian beam excitation used in our experiment, where the Gaussian beam is loosely focused (Supplementary Section S15). The local fields **E**^↔^ and **E**^↕^ are obtained by performing two separate simulations under *x*-polarized (↔) and *y*-polarized (↕) excitations, respectively. Then the local field **E** under any elliptically polarized excitation can be obtained according to Eq. (2). A dipole source is used to simulate the Purcell factor, quantum efficiency and far-field radiation polarization. The Purcell factor is calculated using the power ratio *P*_tot_/*P*_noAnt_, where *P*_tot_ (*P*_noAnt_) is the simulated power emitted from the dipole source in the presence (absence) of the nanoantenna. The quantum efficiency is calculated using the power ratio *P*_rad_/*P*_tot_, where *P*_rad_ is the simulated power emitted from the coupled system. To calculate the far-field polarization, the near-field distribution in a plane slightly below the nanoantenna is simulated, which is then projected to the far field using a far-field projection routine included in the FDTD software.

## Supplementary information

Supplementary Information

## Data Availability

The data that support the findings of this study are available from the corresponding authors upon request.
