# Supplementary material for: Turning a hot spot into a cold spot: polarization-controlled Fano-shaped local-field responses probed by a quantum dot
Source: Light Sci Appl. 2020 Sep 21;9:166. doi: 10.1038/s41377-020-00398-1 (PMC7505841; doi:10.1038/s41377-020-00398-1)
Supplement: Supplementary file 1 — Supplementary Information [file 41377_2020_398_MOESM1_ESM.docx]

Supporting Information for

**Turning a hot spot into a cold spot: Polarization-controlled Fano-shaped local-field responses probed by a quantum dot**

Juan Xia,^1^ Jianwei Tang,^1,2,*^ Fanglin Bao,^3^ Yongcheng Sun,^3^ Maodong Fang,^3^ Guanjun Cao,^3^ Julian Evans,^1^ Sailing He^1,3,4,*^

^1^Centre for Optical and Electromagnetic Research, State Key Laboratory of Modern Optical Instrumentation, National Engineering Research Center for Optical Instrumentation, JORCEP, College of Optical Science and Engineering, Zhejiang University, 310058 Hangzhou, China.

^2^School of Physics, Huazhong University of Science and Technology, Wuhan 430074, China.

^3^Centre for Optical and Electromagnetic Research, ZJU-SCNU Joint Center of Photonics, South China Academy of Advanced Optoelectronics, South China Normal University, Guangzhou 510006, China.

^4^Department of Electromagnetic Engineering, School of Electrical Engineering, Royal Institute of Technology, Stockholm S-100 44, Sweden.

^*^Correspondence: Jianwei Tang ([phystang@hust.edu.cn](mailto:phystang@hust.edu.cn)); Sailing He ([sailing@kth.se](mailto:sailing@kth.se))

## List of contents

**Section S1**. Derivation of Fano lineshapes and fitting by Fano lineshapes

**Section S2**. Experimental setup for optical characterizations

**Section S3**. Negligible z-component of the local fields

**Section S4**. Supplementary simulation results for the theoretical design

**Section S5**. Influence of local-field polarization misalignment on local-field suppression

**Section S6**. Polarization-controlled spectrum and temporal dynamics of the ultrafast local field

**Section S7**. Estimation of the structural parameters of the fabricated QD-loaded nanoantenna

**Section S8**. Fluorescence detection efficiency

**Section S9**. Intrinsic ‘on’-state quantum yield of silica-encapsulated QDs on substrate

**Section S10**. Simulation of the Purcell factor and effective quantum yield for the fabricated sample

**Section S11**. Lifetime shortening attributed to Purcell effect

**Section S12**. Simulation of the local-field distributions for the fabricated QD-loaded nanoantenna

**Section S13**. Simulation for two closely positioned nanoantennas

**Section S14**. Influence of the refractive index of the QD on numerical simulations

**Section S15**. Influence of the plane wave approximation on the numerical simulations

**References for this Supplementary Information**

## Section S1. Derivation of Fano lineshapes and fitting by Fano lineshapes

We assume that the antenna has a resonance under *y*-polarized excitation, which can be approximately described with a Lorentzian resonance $\mathbf{E}^{↕}=Ae^{\text{i}\delta_{\text{A}}}\omega_{0}/\left(\omega-\omega_{0}+\text{i}\gamma/2\right)\hat{\mathbf{u}}$, while under *x*-polarized excitation the response is nonresonant, which can be approximately described with a flat response $\mathbf{E}^{\leftrightarrow}=Be^{\text{i}\delta_{\text{B}}}\hat{\mathbf{u}}$. Here *A*, *B*, $\delta_{\text{A}}$ and $\delta_{\text{B}}$ are real-valued constants in the spectral range of interest, $\omega_{0}$ and $\gamma$ are the resonant frequency and linewidth of the Lorentzian resonance, respectively. If the complete destructive interference (i.e., the minimum local field response) is achieved at a specified frequency $\omega_{\text{s}}$, then according to Eq. (3) the excitation polarization should be

$\theta=\arctan\left(\frac{B}{A\omega_{0}}\left|\omega_{\text{s}}-\omega_{0}+\text{i}\gamma/2\right|\right)$,

.

Under this excitation polarization, according to Eq. (2), the local-field response is

.

The local-field intensity response is

$\left|\mathbf{E}\right|^{2}=\frac{A^{2}\omega_{0}^{2}B^{2}}{A^{2}\omega_{0}^{2}+B^{2}\left[\left(\omega_{s}-\omega_{0}\right)^{2}+\left(\gamma/2\right)^{2}\right]}\frac{\left(\omega-\omega_{\text{s}}\right)^{2}}{\left(\omega-\omega_{0}\right)^{2}+\left(\gamma/2\right)}$.

Using the dimensionless frequency $\Omega\equiv2\left(\omega-\omega_{0}\right)/\gamma$, the Fano asymmetry parameter$q\equiv2\left(\omega_{0}-\omega_{s}\right)/\gamma$ and the quality factor $Q\equiv\omega_{0}/\gamma$, the local-field intensity response can be expressed with the famous Fano formula

$$\left|\mathbf{E}\right|^{2}=D^{2}\frac{\left(\Omega+q\right)^{2}}{\Omega^{2}+1}$$

where $D^{2}=4A^{2}B^{2}Q^{2}\left[4A^{2}Q^{2}+B^{2}\left(q^{2}+1\right)\right]^{-1}$ is a frequency-independent coefficient.

To fit the simulated or experimentally measured spectral dispersion of the local-field response by Fano lineshape, we use the following Fano formula

$$\left|\mathbf{E}\right|^{2}=D^{2}\frac{\left(\Omega+q\right)^{2}}{\Omega^{2}+1}+C^{2}$$

where, compared to the derived Fano formula Eq. , a background term *C*^2^ is added to take into account the local fields that do not interfere due to polarization misalignment. Note that since simulated or measured spectra are in real frequency, to fit simulated or measured spectra the dimensionless frequency Ω and the asymmetry parameter *q* have to be explicitly substituted with $\omega$, $\omega_{0}$, $\omega_{\text{s}}$ and $\gamma$ as $\Omega\equiv2\left(\omega-\omega_{0}\right)/\gamma$ and $q\equiv2\left(\omega_{0}-\omega_{s}\right)/\gamma$. The resonant frequency $\omega_{0}$ and the linewidth $\gamma$ can be determined by fitting the spectral dispersion of the local-field response under *y*-polarized excitation using a Lorentzian lineshape. For any local-field spectrum to be fitted by Fano lineshape, we first directly find the parameters $\omega_{\text{s}}$ and *C*^2^ as the frequency and value of the spectral minimum, respectively. Then only the parameter *D*^2^ needs to be determined by fitting the spectrum with the Fano formula.

## Section S2. Experimental setup for optical characterizations

Optical characterizations of the QD and the QD-loaded nanoantenna are performed using a home-built fluorescence microscopy system (Fig. 1b), with an excitation module with automatic adaptive control of the wavelength, polarization and intensity of the excitation laser light. The excitation laser light is generated and controlled as follows. A broadband pulse laser light is generated by pumping a nonlinear photonic crystal fiber (NLPCF; NKT Photonics, FemtoWHITE 800) with a 750 nm wavelength femtosecond pulse laser (Coherent, Mira 900). This broadband pulse laser light is subsequently filtered by a band-pass filter set to generate a monochromatic pulsed laser light of a desired wavelength. The band-pass filter set is composed of a long-pass edge filter (LPF) and a short-pass edge filter (SPF). The wavelength can be automatically selected or scanned by automatically changing the filter set and tuning the incident angle of the filter. The power of the pulsed laser after the filter set is monitored by splitting part of the power to a power meter (PM) using a beam splitter (BS). The power of the excitation laser light is controlled by a motorized variable neutral density filter (VNDF; Thorlabs, NDL-10C-4). The polarization of the laser light is controlled with a linear polarizer (LP), an achromatic half-waveplate (HWP; Thorlabs, SAHWP05M-1700) and an achromatic quarter-waveplate (QWP; Thorlabs, SAQWP05M-1700) to generate a purely polarized laser light of any elliptical polarization parameters (). Finally, the excitation laser light is focused to the sample by an achromatic focusing objective with low auto-fluorescence (Nikon, S Plan Fluor ELWD 40x, NA 0.6). The residual chromatic aberration is compensated by adjusting the height of the focusing objective for every wavelength point. The diameter of the focal spot is ~ 2 μm. The position of the focal spot is controlled by a pair of beam steering mirrors. This pair of beam steering mirrors and a beam displacement plate (BDP) compensate for beam angle change and beam displacement caused by changing or tuning any optical elements in the beam path during automatic measurements. The mirror-reflection-induced relative phase delay between the *x*- and *y*-polarization components of the excitation light is spectrally measured and corrected with a Soleil-Babinet compensator (Thorlabs, SBC-VIS). The emitted photons from QDs are collected by an achromatic objective with low auto-fluorescence (Nikon, S Plan Fluor ELWD 60x, NA 0.7), passed through a long-pass filter to remove the excitation laser light, and passed through a pinhole (PH) to block stray light, and finally detected by a single photon detector (Picoquant, tau-SPAD). The detector signals are counted by a photon counter (Stanford Research Systems, SR400) to obtain the emission intensity. A monochromator is inserted for analyzing the spectrum of the collected photons. A linear polarizer is inserted for analyzing the polarization of the collected photons. A time-correlated single photon counting module (Picoquant, Picoharp 300) is used for analyzing the fluorescence lifetime.

## Section S3. Negligible *z*-component of the local fields

As shown in Fig. S1, in the *x-y* plane at the same height as the center of the QD, the *z*-components of $\mathbf{E}^{\leftrightarrow}$ and $\mathbf{E}^{↕}$ are negligible.


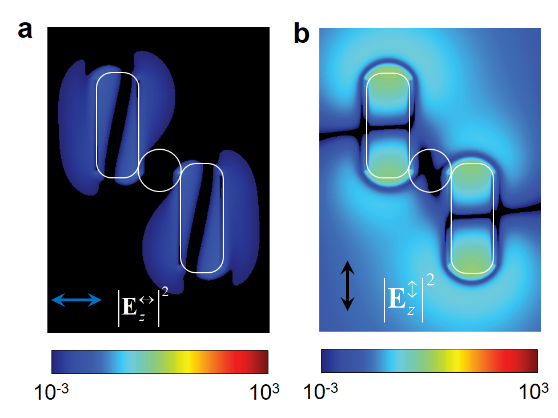


**Fig. S1** **Negligible *z*-component of the local fields under the excitation wavelength of 680 nm.** The distributions of the *z*-component of the local fields under *x*-polarized excitation (**a**) and *y*-polarized excitation (**b**). The color bars are the same as that in Fig. 1. Values smaller than 10^-3^ are represented with black color.

## Section S4. Supplementary simulation results for the theoretical design

With the numerically simulated amplitude and phase dispersions of the local-field responses $\mathbf{E}^{\leftrightarrow}$ and $\mathbf{E}^{↕}$, according to Eq. (3) we can readily calculate the excitation polarization for achieving optimal destructive local-field interference (or local-field suppression) at arbitrarily specified wavelength. Figure S2 shows the calculated excitation polarization parameters as functions of the specified local-field wavelength.

**
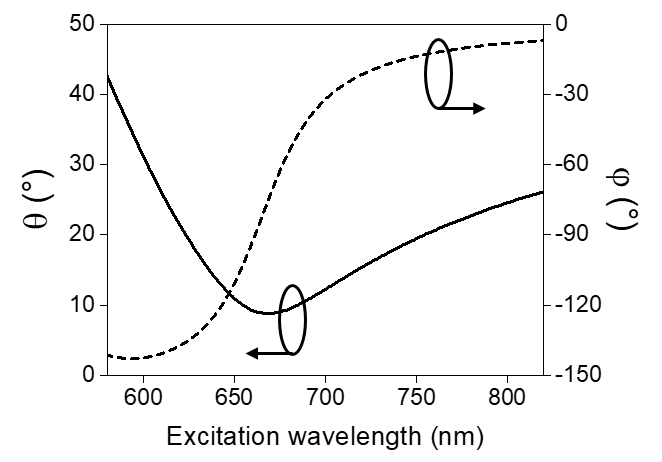
**

**Fig. S2** Calculated excitation polarization parameters as functions of the specified local-field wavelength according to Eq. (3).

The minimum intensity of local-field response as a function of the specified local-field suppression wavelength are plotted in Fig. S3 with a blue curve. The local-field spectral responses plotted in Fig. 1e,f are re-plotted (red curves), with their dips just on the blue curve.

**
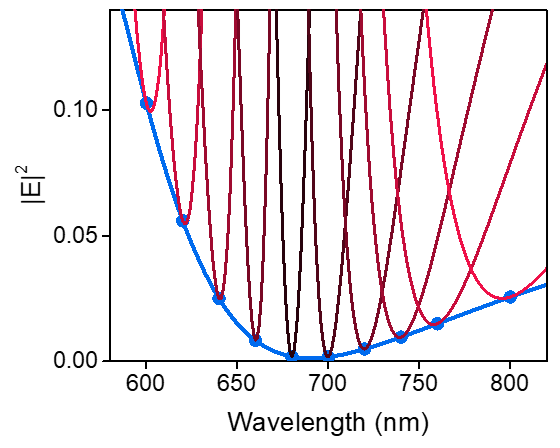
**

**Fig. S3** The minimum local-field response as functions of the specified local-field suppression wavelength (blue curve). The local-field spectral responses plotted in Fig. 1e,f are re-plotted (red curves).

## Section S5. Influence of local-field polarization misalignment on local-field suppression

Figure S3 shows that the minimum local-field responses are small for all the specified wavelengths in the spectral range of interest. However, they are not strictly vanishing, especially for the off-resonant wavelengths (the antenna investigated in Fig. 1 is optimized around the resonant wavelength). This is due to the misalignment between the polarizations of $\mathbf{E}^{\leftrightarrow}$ and $\mathbf{E}^{↕}$. The local-field polarization alignment is optimized around the resonant wavelength. From Fig. 1d, it is clear that for off-resonant wavelengths, the difference between $\psi^{\leftrightarrow}$and $\psi^{↕}$ is the dominant reason for misalignment. Since $\mathbf{E}^{\leftrightarrow}$ and $\mathbf{E}^{↕}$ are nearly linearly polarized according to the nearly vanishing values of $\chi^{\leftrightarrow}$ and $\chi^{↕}$, the minimum local-field response can be approximately expressed as

$\left|\mathbf{E}\right|=2\text{ }\sin\left(\frac{\left|\psi^{\leftrightarrow}-\psi^{↕}\right|}{2}\right)\text{ }\cos\left(\arctan\frac{\left|\mathbf{E}^{\leftrightarrow}\right|}{\left|\mathbf{E}^{↕}\right|}\right)\text{ }\left|\mathbf{E}^{\leftrightarrow}\right|$.

As expected, we can see from Eq. that the minimum local-field response depends on the polarization angle misalignment $\left|\psi^{\leftrightarrow}-\psi^{↕}\right|$ as a sinusoidal function.

Put it another way, we can also say that although there is noticeable misalignment, we can still achieve significant local-field suppression. This indicate that our design is sufficiently robust to tolerate some such misalignment, which is important for successful experimental demonstration. The robustness is attributed to the weak local-field response under *x*-polarized excitation. This has already been indicated by Eq. , which shows that the minimum local-field response also depends on $\left|\mathbf{E}^{\leftrightarrow}\right|$. The smaller the value of $\left|\mathbf{E}^{\leftrightarrow}\right|$, the smaller the minimum local-field response. Figure 1c shows that compared with the strong resonant local-field response $\left|\mathbf{E}^{↕}\right|$, the non-resonant local-field response $\left|\mathbf{E}^{\leftrightarrow}\right|$ is relatively much weaker. So even when there is some polarization misalignment, $\left|\mathbf{E}\right|$ can still be quite small. Otherwise if the local-field response were very strong for both *x*- and *y*- polarized excitations, then even a very small polarization misalignment could lead to a significant value of $\left|\mathbf{E}\right|$, and therefore nearly perfect alignment between the polarizations of $\mathbf{E}^{\leftrightarrow}$ and $\mathbf{E}^{↕}$ would be required to achieve significant local-field suppression.

## Section S6. Polarization-controlled spectrum and temporal dynamics of the ultrafast local field

If local field is excited by a broadband ultrafast pulse as schematically shown in Fig. S4, the spectrum of the local field could be tuned, by controlling the local-field spectral response as demonstrate in Fig. 1e,f. The polarization-controlled tuning of the spectrum of the ultrafast local field is shown in Fig. S5. Then, since coherence is sustained upon local-field response^1-3^, the temporal dynamics of the local field would be accordingly tuned by simply controlling the excitation polarization. The polarization-controlled tuning of the temporal dynamics of the ultrafast local field is accordingly shown in the upper-right inset of every panel in Fig. S5.


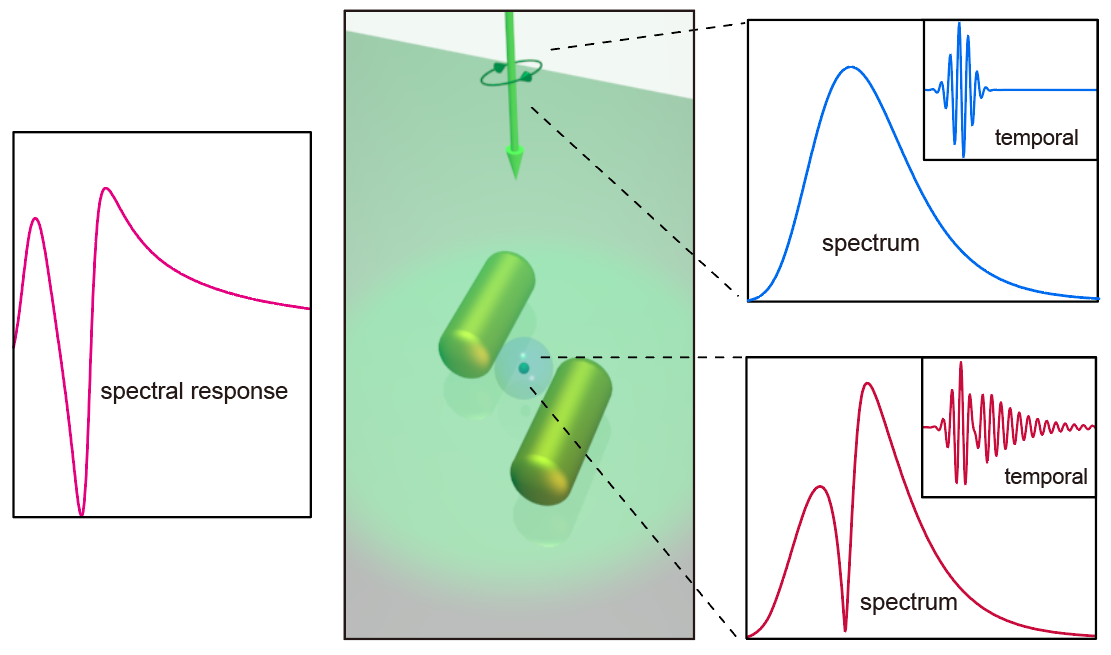


**Fig. S4 Spectrum and temporal dynamics of local field excited by a broadband ultrafast pulse**. The upper-right panel shows the spectrum and temporal dynamics of the incident ultrafast pulse, which features a broadband spectrum and a short pulse width. The left panel shows the local-field spectral response of the nanoantenna, which can be controlled by excitation polarization as demonstrated in Fig. 1e,f. The lower-right panel shows the spectrum and temporal dynamics of the resultant local field, which are determined by both the incident pulse and the local-field spectral response of the antenna.


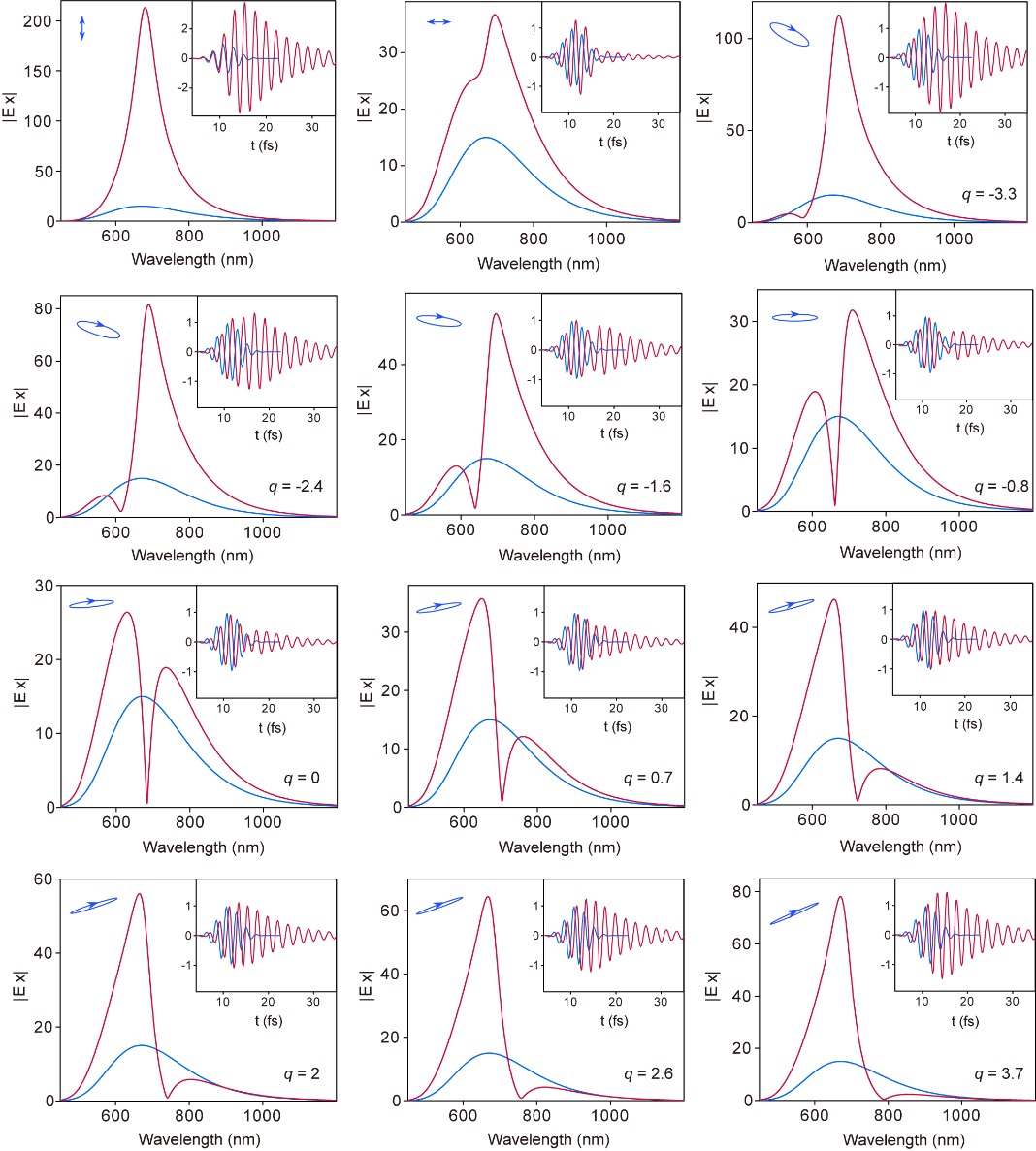


**Fig. S5** **Polarization-controlled spectrum and temporal dynamics of the ultrafast local field.** In each panel, the blue curves are the spectrum and time dynamics of the incident pulse; the red curves are the spectrum and time dynamics of the generated local field. The incident pulse is the same for every panel, only its excitation polarization (denoted in the upper-left corner) is controlled for each panel. The excitation polarization in each panel just corresponds to one used in Fig. 1, the denoted *q* parameter of the Fano fit of the local-field response can be used for correspondence.

## Section S7. Estimation of the structural parameters of the fabricated QD-loaded nanoantenna

The structure parameters of the QD-loaded nanoantenna are sketched in Fig. S6a. The structure parameters of the fabricated sample (Fig. 2a) are estimated as follows, which are then used for simulations. The diameters of the GNRs G1 (*d*_1_) and G2 (*d*_2_) and the silica-encapsulated QD (*d*) are determined according to their heights measured by AFM topographic imaging (see Fig. S6b,c). This way, *d*_1_, *d*_2_ and *d* are determined to be 32 nm, 34 nm and 31 nm, respectively. The lateral distances between the height peaks of the GNRs and the QD, i.e., the values of *g*_1_+*d*_1_/2 and *g*_2_+*d*_2_/2, can be read from the AFM profiles (Fig. S6c). Since the GNR diameters *d*_1_ and *d*_2_ are already known, then *g*_1_ and *g*_2_ can determined. This way, *g*_1_ and *g*_2_ are determined to be 16 nm and 20 nm, respectively. That is, the gap width is 36 nm and the position of the QD deviates slightly from the center of the gap by 2 nm towards the GNR G1. The parameters *δ*_1_ and *δ*_2_ are estimated from the AFM topographic image to be 0 nm. Then the lengths of the GNRs G1 (*l*_1_) and G2 (*l*_2_) are the only parameters undetermined. The length of a GNR cannot be accurately determined directly from the AFM image. But it can be determined with the aid of the scattering spectrum of the GNR, since with the diameter already known from AFM imaging, the resonant wavelengths of the scattering spectrum would only depend on the length of the GNR. So we determine the length of the GNRs according to the darkfield scattering spectra measured *before* they are assembled (Fig. 2b). Specifically, we fit the simulated scattering spectrum of the GNR to the measured darkfield scattering spectrum, with the length of the GNR as the only free parameter (see Fig. 2b for the fitting results). This way, the length of GNRs *l*_1_ and *l*_2_ are determined to be 78 nm and 80 nm, respectively. Interestingly, the line widths of the scattering spectra are also fitted very well, which indicates that the loss of gold here is well described by the data taken from Johnson and Christy^4^.


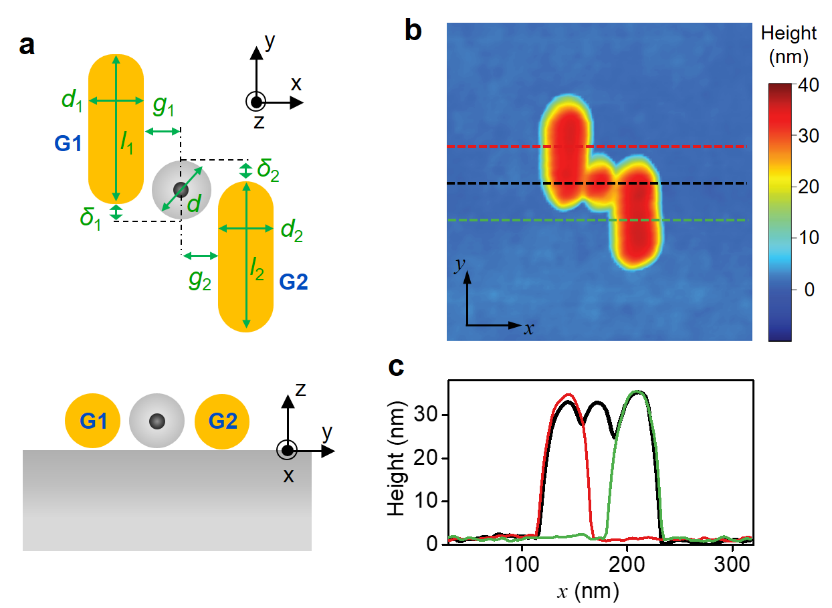


**Fig. S6** **Determination of structure parameters of the fabricated QD-loaded nanoantenna**. (**a**) Sketch of the structure parameters of the QD-loaded nanoantenna, viewed from +z direction (upper panel) and +x direction (lower panel). (**b**) AFM topographic image. (**c**) Profiles along the dashed lines shown in panel (b).

## Section S8. Fluorescence detection efficiency

The detection efficiency of QD fluorescence is determined as follows. First, the product of optical transmittance (*T*) and detector efficiency ($\xi^{\text{SPAD}}$) is probed by a laser beam of known power. Specifically, a linearly polarized laser beam of 808 nm is loosely focused on to the glass substrate and collected by the objective (NA = 0.7). The power of the laser beam is measured at the substrate by a power meter. Then the laser beam is attenuated with ND filters whose transmittances are measured in advance, so that the power of the laser beam is suitable to be detected with the single photon detector. Then the laser beam, whose power is weak and known, is collected by the optical system and detected by the single photon detector. From the photon counting rate, the product $T\cdot\xi^{\text{SPAD}}$ can be easily calculated. For the *x*-polarized laser beam, $T\cdot\xi^{\text{SPAD}}$ is measured to be 10.1%, while for the *y*-polarized laser beam, $T\cdot\xi^{\text{SPAD}}$ is measured to be 9.8%. The polarization dependence is very weak, so we simply neglect the polarization dependence and take the polarization-averaged value 9.95% as the value of $T\cdot\xi^{\text{SPAD}}$. In our experiment, the long-pass filter used to cut the excitation wavelength also spectrally cut part of the emission. This is taken into account by separately measuring the transmission of the filter $T_{\text{filter}}$ directly using the emission of our QD, which is measured to be 68%.

Second, the collection efficiency of the objective for horizontal and vertical dipole emitters are calculated using FDTD simulation. An objective can only collect light within the solid angle defined by its numerical aperture (NA). For the loosely focused laser used above to probe the detection efficiency of the optical system, the light completely falls into the NA of our objective (NA = 0.7). But for a dipole emitter, the objective can only collect part of its radiation. We define the collection efficiency of the objective as $\xi^{\text{obj}}=P_{\text{obj}}/P_{\text{tot}}$, where $P_{\text{obj}}$ is the radiation power integrated in the solid angle defined by the NA of the objective, $P_{\text{tot}}$ is the total radiation power from the dipole emitter. Note that the transmittance of the objective is not taking into account in the collection efficiency, since it has already been included in the detection efficiency for laser beam. The collection efficiency depends on the dipole orientation. Any dipole orientation can be decomposed into the horizontal component and the vertical component. Here the collection efficiency for horizontal and vertical dipoles are numerically calculated by integrating power in the far field within the solid angle defined by the NA of the objective. Specifically, a dipole source is placed 15 nm above the substrate and then the near field distribution in a plane slightly below the substrate is obtained using FDTD simulation. Then electric field in the far field are calculated by performing a far field projection. Then the power over the solid angle defined by the NA of the objective is calculated by integrating the Poynting vector. Finally, for our objective with NA = 0.7, the collection efficiency for the horizontal dipole $\xi_{\parallel}^{\text{obj}}$ is calculated to be 14.4%, and the collection efficiency for the vertical dipole $\xi_{\perp}^{\text{obj}}$ is calculated to be 2.83%.

Third, the detection efficiency for arbitrarily oriented 2D dipole emitter can then be calculated. The orientation of a 2D dipole emitter is measured through polarization analysis of its far-field emission. The orientation of a 2D dipole is represented by the orientation angle Θ and Φ of its dark axis as schematically shown in Fig. S7. Namely, Θ = 0 means the dark axis is normal to the substrate and the 2D dipole plane is horizontal. The azimuthal angle Ψ does not influence the detection efficiency since the transmittance of our optical system is nearly polarization-independent. The 2D dipole can decomposed into two non-coherent linear dipoles as , where $\mu_{\parallel}$ denotes a horizontal linear dipole and $\mu_{\Theta}$denotes a linear dipole with an inclination angle Θ with the horizontal plane. The tilted dipole $\mu_{\Theta}$ can be further decomposed into a horizontal dipole and a vertical dipole. So the collection efficiency of the 2D dipole can be expressed as

For an objective with NA=0.7, the emission collected from a horizontal linear dipole is linearly polarized (the unpolarized component collected by an objective with NA below 0.8 can be neglected), while the emission collected from a vertical linear dipole is completely unpolarized. So if the emission collected by the objective is analyzed by a linear polarizer (i.e., the intensity as a function of the polarization angle of the polarizer is measured), there will be a minimum when the polarization angle of the polarizer is Φ and a maximum when the polarization angle of the polarizer is Φ+90°. The minimum and maximum collection efficiency with the presence of the linear polarizer can be expressed as and , respectively. The degree of linear polarization (DOLP), which is defined as $\alpha\equiv\left(\xi_{\max}-\xi_{\min}\right)/\left(\xi_{\max}+\xi_{\min}\right)$, can be experimentally obtained from polarization analysis. Then according to Eq. , the orientation-dependent collection efficiency can be expressed as

$\xi^{\text{obj}}=\frac{\xi_{\parallel}^{\text{obj}}}{1+\alpha\left(1-\xi_{\perp}^{\text{obj}}/\xi_{\parallel}^{\text{obj}}\right)}$.

Using the simulated values of $\xi_{\parallel}^{\text{obj}}$ and $\xi_{\perp}^{\text{obj}}$ for our objective with NA=0.7, we can get$\xi^{\text{obj}}=\left(1+0.8\alpha\right)^{-1}\cdot14.4\%$.

Considering all the issues discussed above, the detection efficiency of QD emission can be expressed as

$\xi=T\cdot\xi^{\text{SPAD}}\cdot T_{\text{filter}}\cdot\xi^{\text{obj}}$.

Putting all the values into the expression, we get

$\xi=\left(1+0.8\alpha\right)^{-1}\cdot0.97\text{\%}$,

which depends on the orientation of the QD. For a QD with a horizontal bright plane, the DOLP *α* = 0, so $\xi=0.97\text{\%}$.


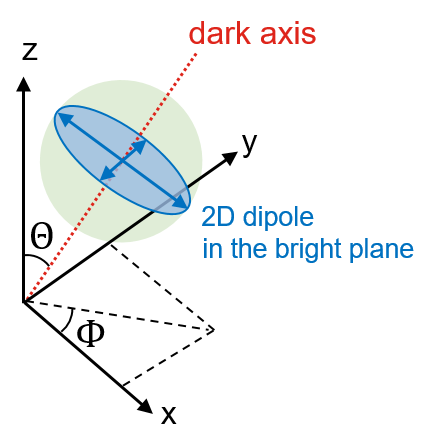


**Fig. S7** **Orientation of a 2D dipole representing the QD**. The 2D dipole can decomposed into two noncoherent linear dipoles, one horizontal and the other with an inclination angle Θ with the horizontal plane.

## Section S9. Intrinsic ‘on’-state quantum yield of silica-encapsulated QDs on substrate

Knowing the detection efficiency for the fluorescence from QD, the intrinsic ‘on’-state quantum yield of QD can be estimated by measuring fluorescence from a single QD as a function of the excitation power. In our experiment, we use a pulsed laser with a repetition rate of 2MHz to excite a silica-encapsulated QD whose bright plane is nearly parallel to the substrate surface. For every excitation power, the photon counting data is recorded by the TCSPC system, then the time trajectory of the photon counting rate can be obtained by post-processing, from which the ‘on’ state of the QD can be identified and the ‘on’-state counting rate can be obtained and plotted as shown in Fig S7a. When the power of the pulsed laser becomes strong enough, we can saturate the probability of generating at least one exciton in a pulse period, and then the photon counting rate saturates. Fig. S8b and Fig. S8c show the ‘on’-state lifetime curves at weak excitation power P1 and saturated excitation power P2. Here the lifetime curves are called ‘on’-state lifetime curves because the data for plotting the lifetime curves are extracted from the ‘on’-state periods. The lifetime curves indicate that multi-exciton emissions are not significant even for saturated excitation. Therefore, we can fit the experimental data in Fig. S8a with an exponential saturation curve $I\left(P\right)=I_{\text{sat}}\left(1-e^{-P/P_{\text{sat}}}\right)$, where $I_{\text{sat}}$ is the saturation counting rate and $P_{\text{sat}}$ is the saturation excitation power^5^. Through the saturation fitting (red curve in Fig S7a), the saturation counting rate is estimated to be ~19000$\pm$3000 counts per second. Taking into account the detection efficiency 0.97% (see Supplementary Section S9) and the repetition rate of the pulsed laser 2MHz, the quantum yield can be estimated to be 98%$\pm$15%.

The measured QD is a single QD as indicated by the binary blinking behavior in the time trajectory of the photon counting rate (Fig. S8d). Note that the QD for saturation curve measurement is not the QD coupled to the antenna. This is because saturation curve measurements almost always deteriorate the fluorescence quality of the QD. If not photobleached, the binary blinking behavior of the QD would be significantly deteriorated due to the appearance of grey states, as shown in Fig. S8e. Although not the same QD, we can still infer that the QD coupled to the antenna has similar near-unity intrinsic ‘on’-state quantum yield by comparing their fluorescence lifetimes. The lifetime of the QD to be coupled to the antenna is ~246$\pm$12ns, which is similar to the lifetime of the QD for saturation curve measurement. This indicates that there is no significant quenching channels to reduce the quantum yield, since significant quenching should be accompanied by lifetime shortening.

The near-unity intrinsic ‘on’-state quantum yield measured in this work is consistent with references, where the intrinsic quantum yield of single core/shell QDs in their ‘on’ state were reported to be near unity in colloidal solution^6-8^, or in polymer ^9,10^, or in ambient condition on the substrate ^11-13^. It is worth noting that in order to get a near-unity quantum yield, the QD should be well protected from being quenched by the environment. In the references reporting near-unity quantum yield, the QDs are either in colloidal solution^6-8^, or in polymer^9,10^, or are protected by a thick shell^11-13^. In our work, the purchased CdSeTe/ZnS core-shell QDs are encapsulated with a thick silica shell. This thick silica shell protests the QD from being quenched by the environment. So the QD can have near-unity quantum yield on the substrate. The quenching due to the contact with substrate can be considered as nonradiative energy transfer to surface recombination centers generated due to the contact with the substrate. With the thick silica shell, the surface recombination centers are separated with the center of the QD by ~15 nm. This should make the nonradiative energy transfer rather slow.

The absence of quenching by the substrate can also be inferred from lifetime measurements, since significant quenching should be accompanied by lifetime shortening. The QDs with silica shell and QDs without silica shell both have a similar lifetime ~160 ns when they are in the colloidal solution. When they are transferred onto the substrate, the QDs with silica shell and the QDs without silica shell have rather different lifetimes. The lifetimes of the QDs with silica shell is measured to be over 200 ns, which is even longer than their lifetimes in the colloidal solution. The absence of lifetime shortening indicates that quenching due to the contact with substrate is unlikely for the QDs with silica shell. While for the QDs without silica shell, the lifetimes are measured to be in the range 50-100 ns, which are significantly shorter than their lifetimes in the colloidal solution. The lifetime shortening indicates that there may indeed be some quenching by surface recombination centers that are generated due to the contact with the substrate, which is reasonable since in absence of the silica shell the surface recombination centers are close to the core of the QD.

Based on all the considerations above, it is reasonable to assume the intrinsic ‘on’-state quantum yield of our QD to be unity.


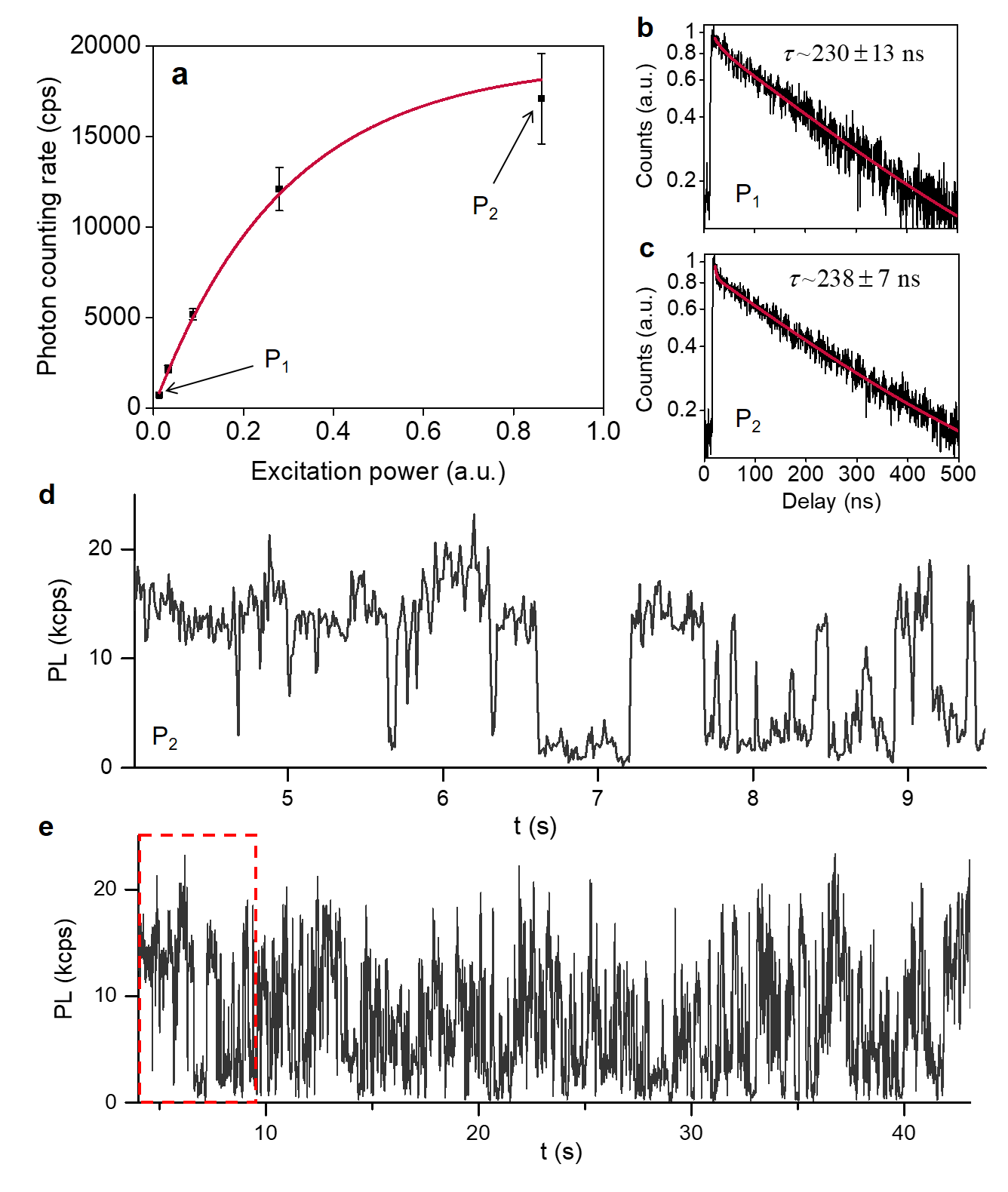


**Fig. S8** **Determination of the intrinsic ‘on’-state quantum yield of a silica-encapsulated QD.** (**a**) Detected photon counts from the QD as a function of the excitation power. The red curve is a fit by an exponential saturation curve. (**b**,**c**) The ‘on’-state lifetime curves measured at the excitation powers P_1_ (b) and P_2_ (c) denoted in panel a. The red curves are fits by exponential decay functions. The lifetimes obtained from exponential decay fitting are denoted beside the curves. (**d**,**e**) The time trajectory of the photon counting at the excitation power P_2_. Panel d is for the beginning time range from ~4s (the excitation starts at ~4 s) to 9.5s. Panel e is for the time range from ~4s to ~43 s. The red dashed frame indicated in panel e shows the time range for panel d.

## Section S10. Simulation of the Purcell factor and effective quantum yield for the fabricated sample

The QD is weakly excited throughout our work, so the probability of excitation of biexcitons or multiexcitons can be neglected and only the decay of monoexcitons needs to be considered in our analysis. The intrinsic nonradiative decay is neglected since the intrinsic quantum yield of the QD in the ‘on’ state is near unity (see Supplementary Section S9). We consider the transition dipole of QD as a 2D-dipole in the bright plane of the QD^14^. The bright plane of the QD is made nearly horizontal by rotating and rolling the silica-encapsulated QD with an AFM tip. So the 2D-dipole can be simply treated as a noncoherent combination of an *x*-oriented linear dipole and a *y*-oriented linear dipole, both with a decay rate of $\gamma^{0}$ and a population probability of 0.5. Here $\gamma^{0}$ is the intrinsic decay rate of the 2D-dipole. After coupling with the nanoantenna, due to the Purcell effect, the decay rate of the *x*-oriented linear dipole is enhanced by a factor of $f_{x}$ to be $\gamma_{x}=f_{x}\gamma^{0}$, while the decay rate of the *y*-oriented linear dipole is enhanced by a factor of $f_{y}$ to be $\gamma_{y}=f_{y}\gamma^{0}$. So the decay rate of the 2D-dipole is

$$\gamma_{\text{2D}}=0.5\gamma_{x}+0.5\gamma_{y}=0.5\left(f_{x}+f_{y}\right)\gamma^{0},$$

and the Purcell factor for the 2D-dipole is

$$f_{\text{2D}}=0.5\left(f_{x}+f_{y}\right).$$

The Purcell factors $f_{x}$ and $f_{y}$ are numerically obtained as a function of emission wavelength (Fig. S9a). With the numerically calculated $f_{x}$ and $f_{y}$, the Purcell factor $f_{\text{2D}}$ can then be obtained using Eq. . The Purcell factor $f_{\text{2D}}$ as a function of emission wavelength is plotted as solid black curve in Fig. S9a.

To measure the absorption spectra of the uncoupled QD or the QD coupled with the nanoantenna using photoluminescence excitation (PLE) spectroscopy, the knowledge of the collection efficiency for QD emission is required. Here, the collection efficiency is a relative efficiency normalized to the collection efficiency for a horizontally oriented linear dipole. In this study, the collection efficiency for QD emission is influenced by two factors: dipole orientation and antenna loss.

Since the bright plane of the QD is made nearly horizontal, the photon collection efficiency of the uncoupled QD is the same as the photon collection efficiency for a horizontally oriented linear dipole. After the plasmonic nanoantenna is assembled to couple with the QD, the emission of the QD couples to the plasmonic modes. This leads to two consequences. First, the orientation of the effective dipole changes. However, since the effective dipoles of the plasmonic modes are still horizontal, this shall not influence the photon collection efficiency. Second, the effective quantum yield reduces, which reduces the photon collection efficiency. Due to the material loss of the plasmonic nanoantenna, the quantum yield of the *x*-oriented linear dipole $\eta_{x}$ and the quantum yield of the *y*-oriented linear dipole $\eta_{y}$ are less than 100%. The quantum yield of the 2D-dipole can be expressed as

$$\eta_{2\text{D}}=\frac{\gamma_{x}\eta_{x}+\gamma_{y}\eta_{y}}{\gamma_{x}+\gamma_{y}}=\frac{f_{x}\eta_{x}+f_{y}\eta_{y}}{f_{x}+f_{y}}.$$

The Purcell factors $f_{x,y}$ and the quantum yields $\eta_{x,y}$ are numerically obtained as a function of emission wavelength, as shown in Figs. S9a and b, respectively. With the numerically calculated $f_{x,y}$ and $\eta_{x,y}$, the quantum yield $\eta_{2\text{D}}$ can then be obtained using Eq. . The quantum yield $\eta_{2\text{D}}$ as a function of emission wavelength is plotted as solid black curve in Fig. S9b. At the emission wavelength (~808 nm) of the QD, the quantum yield is ~56%.


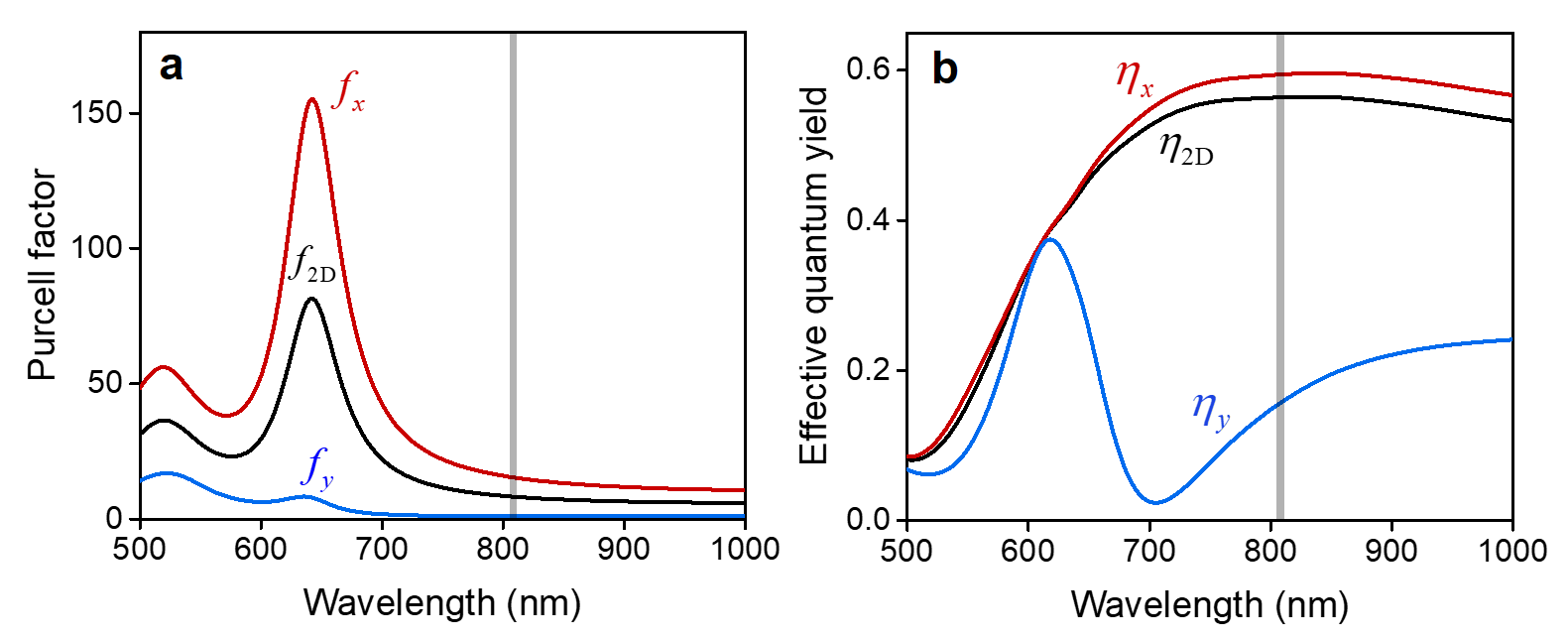


**Fig. S9** **Dipole-orientation-dependent Purcell factors and quantum yields.** (**a**) Numerically calculated Purcell factors $f_{x}$ and $f_{y}$ as a function of emission wavelength. The black curve is the Purcell factor for the 2D-dipole $f_{2\text{D}}$ calculated according to Eq. . The grey vertical line indicates the emission wavelength of the QD used in this work. (**b**) Numerically calculated quantum yields $\eta_{x}$and $\eta_{y}$ as a function of emission wavelength. The black curve is the quantum yield of the 2D-dipole $\eta_{2\text{D}}$ calculated according to Eq. . The grey vertical line indicates the emission wavelength of the QD used in this work.

## Section S11. Lifetime shortening attributed to Purcell effect

Lifetime shortening or decay rate enhancement must be due to additional decay channels generated upon coupling to the metallic nanostructure. The additional decay channels may be any of the following three types. The first possible decay channel is electromagnetic energy transfer from QD to the plasmonic modes of the nanoantenna. This decay channel leads to the enhancement of local density of photonic states (LDOS), i.e., Purcell effect. The second possible decay channel is nonradiative energy transfer to surface recombination centers that are generated due to the contact with the metal surface. The third possible decay channel is carrier tunneling from the QD to metal.

Carrier tunneling is unlikely in our experiment, since we have encapsulated the QD with an insulating silica shell of more than 10 nm thickness (the diameter of the silica-encapsulated QD is ~31 nm, while the diameter of the QD itself is less than 10 nm). It’s worth noting that even the organic ligands on the surface of QD has been proven to provide enough barrier to make the tunneling of carriers to the metal very slow ^15^.

Nonradiative energy transfer to surface recombination centers generated due to the contact with the metal surface can also be neglected owing to the thick silica shell. With the thick silica shell, the surface recombination centers are separated with the center of the QD by ~15 nm. This should make the nonradiative energy transfer quite slow. Indeed, we have observed that the lifetime of QD with silica shell has a longer lifetime on substrate (>200 ns) than in colloidal solution (~160 ns). This indicates that the surface recombination centers generated due to contact with substrate does not lead to observable nonradiative energy transfer. For comparison, the lifetime of QD without silica shell has a significantly shorter lifetime on substrate (<100 ns) than in colloidal solution (~160 ns), which may be attributed to nonradiative energy transfer to the surface recombination centers that are generated due to the contact with the substrate.

The decay rate enhancement observed in our experiment can be well attributed to electromagnetic energy transfer from QD to the plasmonic modes of the nanoantenna, i.e., Purcell effect. First, the altered fluorescence polarization agrees well with the electromagnetic simulation (as shown in Fig. 2d). This indicates that the QD has indeed decayed to the plasmonic modes. If the lifetime reduction were due to quenching, then the radiation polarization would not have be governed by the antenna. Second, the experimentally observed 7-fold decay rate enhancement can be well-explained by the electromagnetically simulated Purcell factor. The electromagnetic simulation shows that although the emission wavelength 808 nm is far detuned from the resonance of the nanoantenna, there is still a significant Purcell factor of ~8.5 for a horizontal 2D-dipole (see Fig. S9a; at the resonance the Purcell factor for a horizontal 2D-dipole would be ~80).

## Section S12. Simulation of the local-field distributions for the fabricated QD-loaded nanoantenna

At the resonant wavelength of the nanoantenna (645 nm), the local-field distributions of $\mathbf{E}^{\leftrightarrow}$ and $\mathbf{E}^{↕}$ are plotted in Fig. S10a and Fig. S10b, respectively. The polarization distributions of $\mathbf{E}^{\leftrightarrow}$ and $\mathbf{E}^{↕}$ are plotted in Figs. S10c-f, from which we see that despite the markedly different overall distributions, $\mathbf{E}^{\leftrightarrow}$ and $\mathbf{E}^{↕}$ have similar polarizations in the nanogap. This nontrivial feature is also indicated by the instantaneous electric displacement vectors in Figs. S10a,b. So efficient interference between the local field excited by the *x*-component of the incident light and the local field excited by the *y*-component of the incident light can take place in the nanogap according to Eq. (2). When the excitation polarization satisfies the destructive interference conditions given by Eq. (3), the local-field distribution shows a cold spot in the nanogap (Fig. S10g), which indicates significant local-field suppression. When the excitation polarization is orthogonal, the interference becomes constructive and the local-field distribution shows a hot spot in the nanogap (Fig. S10h).

Comparing between Fig. S10 (for excitation wavelength of 645 nm) and Fig. S11 (for excitation wavelength of 725 nm), we see that the polarizations of $\mathbf{E}^{\leftrightarrow}$ and $\mathbf{E}^{↕}$ in the gap region are more similar for the excitation wavelength of 725 nm than for the excitation wavelength of 645 nm, which explains the higher suppression factor at 725 nm than at 645 nm (Fig. 4b). Simulations show that strong local-field suppression can be achieved at different heights as shown in Fig. S12, *i.e.*, the cold spot is a three-dimensional nano spot.


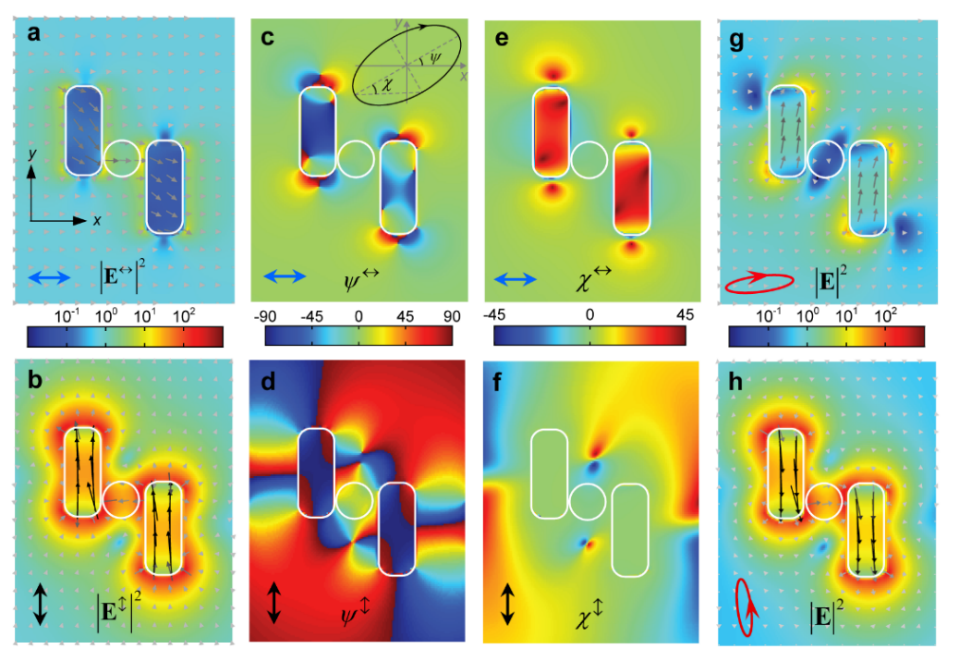


**Fig. S10** **Local-field suppression and enhancement under the excitation wavelength of 645 nm.** (**a**,**c,e**) Numerically calculated distributions of the electric field intensities and electric field polarizations ($\psi^{\leftrightarrow}$ and $\chi^{\leftrightarrow}$) under *x*-polarized excitation. (**b**,**d**,**f**) Numerically calculated distributions of the electric field intensities $\left|\mathbf{E}^{↕}\right|^{2}$ and electric field polarizations ($\psi^{↕}$ and $\chi^{↕}$) under *y*-polarized excitation. (**g**,**h**) Numerically calculated distribution of electric field intensity $\left|\mathbf{E}\right|^{2}$ (color map) and instantaneous electric displacement vectors (arrow map) under elliptically polarized excitation (shown in the bottom-left corner) for optimal local-field suppression (g) or optimal local-field enhancement (h). The arrow maps in (a), (b), (g) and (h) depict the distributions of instantaneous electric displacement vectors.


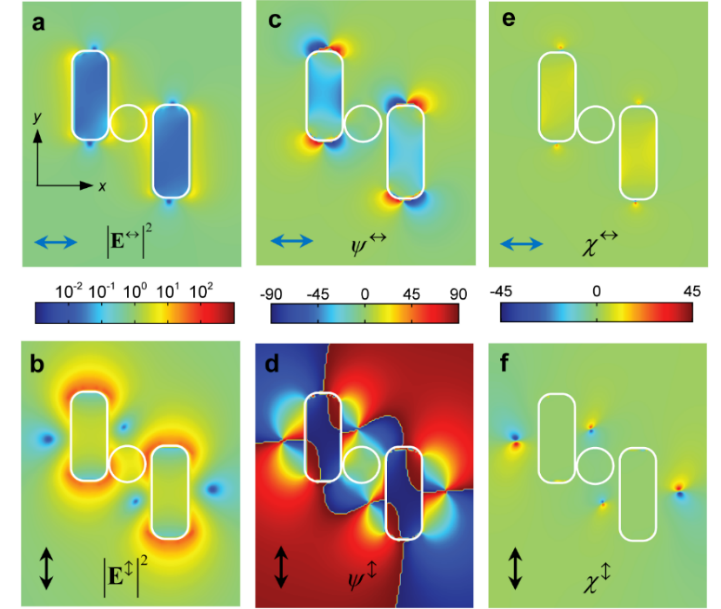


**Fig. S11 Polarization-dependent local-field distributions under excitation at the wavelength of 725 nm.** (**a**,**c,e**) Numerically calculated distributions of the electric field intensities $\left|\mathbf{E}^{\leftrightarrow}\right|^{2}$ and electric field polarizations ($\psi^{\leftrightarrow}$ and $\chi^{\leftrightarrow}$) under *x*-polarized excitation. (**b**,**d**,**f**) Numerically calculated distributions of the electric field intensities $\left|\mathbf{E}^{↕}\right|^{2}$ and electric field polarizations ($\psi^{↕}$ and $\chi^{↕}$) under *y*-polarized excitation. The plotting plane is the x-y plane at the same height as the center of the QD.

**
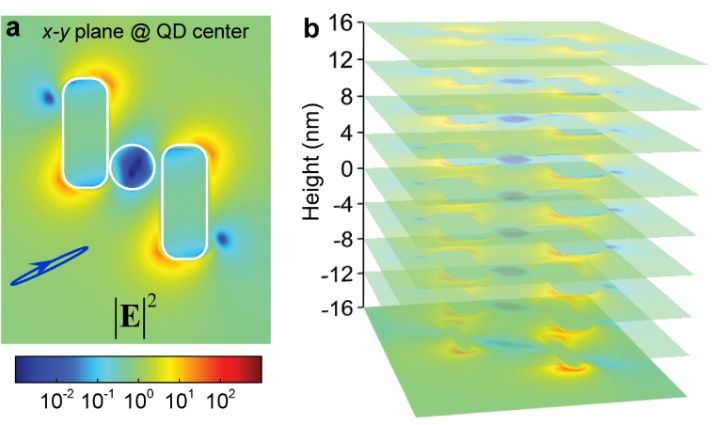
**

**Fig. S12 Local-field suppression at different height under excitation at the wavelength of 725 nm.** (**a**) Local-field distribution in the *x-y* plane at the same height as the center of the QD. (**b**) Local-field distributions in the *x-y* planes at different heights relative to the center of the QD. The excitation polarization is denoted by the polarization ellipse in (a). All the local-field distributions share the color bar in (a).

## Section S13. Simulation for two closely positioned nanoantennas

Here two closely positioned antennas are simulated to demonstrate a high contrast ratio between the antennas. For higher spatial resolution, smaller sized antennas are used here, as shown in Fig. S13. The antenna is composed of two identical GNRs with a diameter of 10 nm. The gap width is 10 nm. The optimal excitation enhancement factor is ~320 and the optimal excitation suppression factor is ~0.0167, the wavelength-specified dynamic range of local-field control is ~19000.

If two such identical antennas are closely positioned in a subwavelength region with orthogonal orientations (Fig. S14), a high contrast ratio between the antennas can then be achieved. If we use the excitation polarization denoted in the upper-left corner of Fig. S14a, we can get a field distribution where there is a hot spot at QD2 while a cold spot at QD1, with a spatial contrast ratio ~21000; if we change the excitation polarization to that denoted in upper-left corner of Fig. S14b, we can get a field distribution where there is a hot spot at QD1 while a cold spot at QD2, with a spatial contrast ratio ~20000. It is interesting that for such a setup, the spatial contrast ratio is roughly the value of the wavelength-specified dynamic range of local-field control for each antenna. This is expected and somehow imply the significance of the wavelength-specified dynamic range of local-field control.

Note that the excitation polarization found for optimal excitation suppression of Q1 or Q2 is slightly different from that when there is only one antenna. This is attributed to the coupling between the antennas, as can be observed from the field distributions. This indicates that local-field interference works well even in the presence of some coupling between antennas.


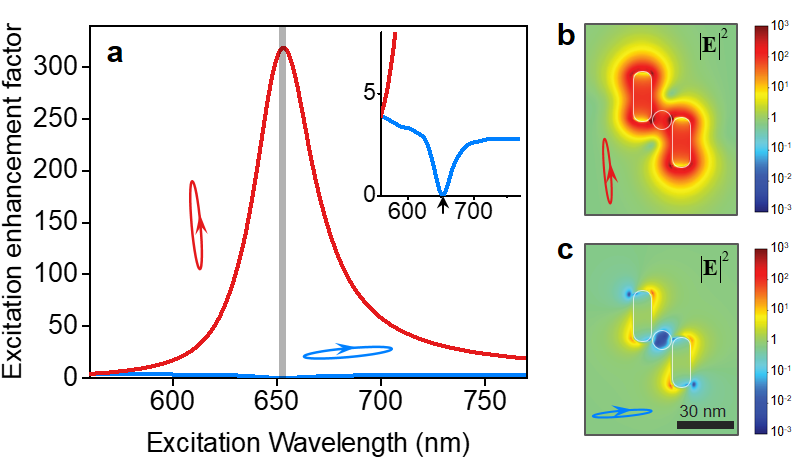


**Fig. S13 Simulation results for a smaller nanoantenna.** (**a**) Excitation enhancement spectra when excited with the excitation polarizations denoted, which are found for optimal excitation enhancement (red) and optimal excitation suppression (blue) at the specified excitation wavelength of 653 nm, respectively. The upper-right inset re-plots the spectra with zoomed y axis. (**b**) The field distribution at 653 nm when excited with excitation polarization denoted, which is found for optimal excitation enhancement. (**c**) The field distribution at 653 nm when excited with excitation polarization denoted, which is found for optimal excitation suppression.


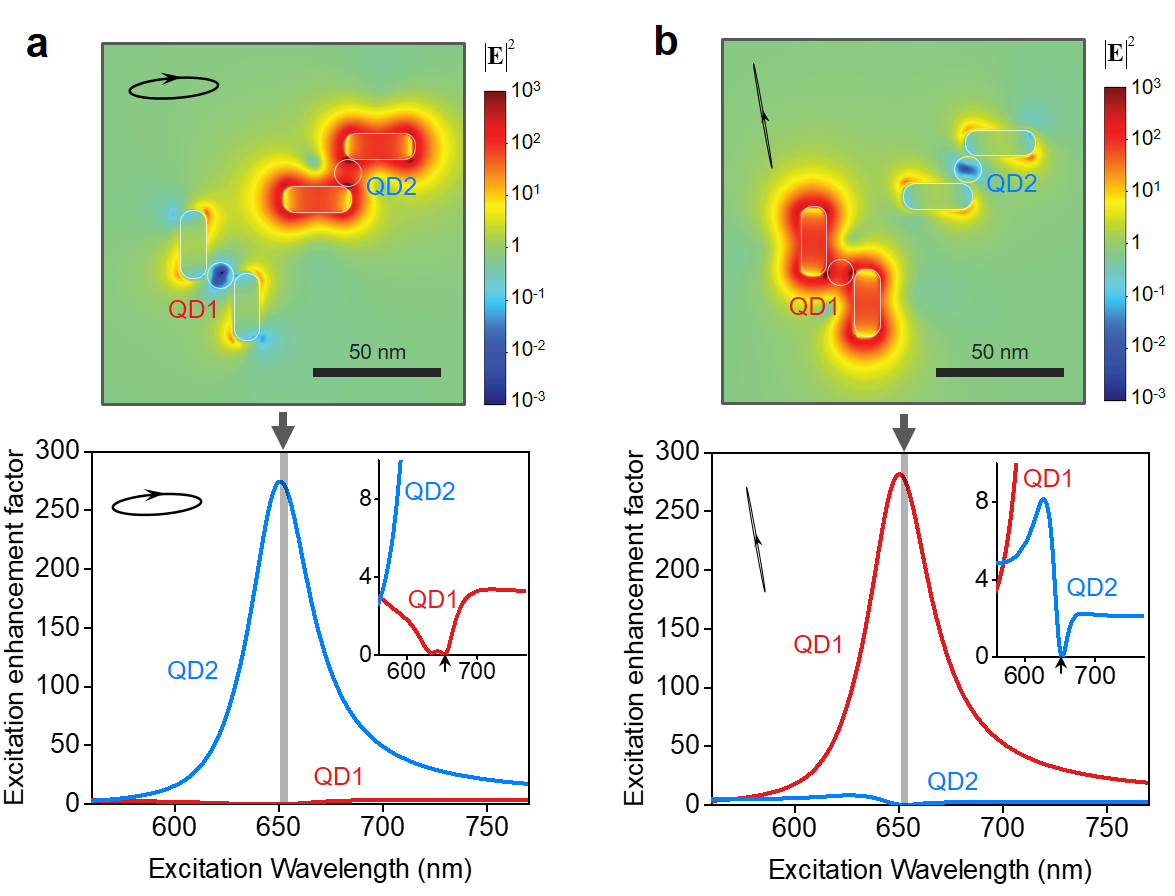


**Fig. S14** **Simulation results for closely positioned nanoantennas.** (**a**) Field distribution (upper panel) and excitation enhancement spectra (lower panel) when excited with the excitation polarization denoted, which is found for optimal excitation suppression of QD1 at the specified excitation wavelength of 653 nm. The field distribution is at 653 nm. (**b**) Field distribution (upper panel) and excitation enhancement spectra (lower panel) when excited with the excitation polarization denoted, which is found for optimal excitation suppression of QD2 at the specified excitation wavelength of 653 nm. The field distribution is at 653 nm.

## Section S14. Influence of the refractive index of the QD on numerical simulations

The silica-encapsulated QD is simply modelled as a homogeneous and isotropic silica sphere. For all the simulations, the dielectric presence of this silica sphere is taken into account. Note that modelling the silica-encapsulated QD simply as a homogeneous and isotropic silica sphere while neglecting the higher refractive index of the pristine QD itself does not influence the simulated excitation enhancement factor, as shown in Fig. S15a. This is because the excitation enhancement factor is defined as the ratio $f_{\text{exc}}=\left|\mathbf{E}_{\text{Ant}}\right|^{2}/\left|\mathbf{E}_{\text{noAnt}}\right|^{2}$. Although $\left|\mathbf{E}_{\text{Ant}}\right|^{2}$and $\left|\mathbf{E}_{\text{noAnt}}\right|^{2}$ significantly decrease when the high refractive index of the QD itself is taken into account (as shown in Fig. S15b), their ratio barely changes. Similarly, the simulation of Purcell factor is not influence by not considering the refractive index of the pristine QD itself either, as shown in Fig. S15c. Because the Purcell factor is defined as the ratio $f_{\text{Purcell}}=P_{\text{tot}}/P_{\text{noAnt}}$, where *P*_tot_ is the total power emitted from the dipole source in the presence of the nanoantenna, $P_{\text{noAnt}}$ is the power emitted from the dipole source in the absence of the nanoantenna. Although $P_{\text{tot}}$ and $P_{\text{noAnt}}$are influenced by the high refractive index of the QD (as shown in Fig. S15d), their ratio barely changes.


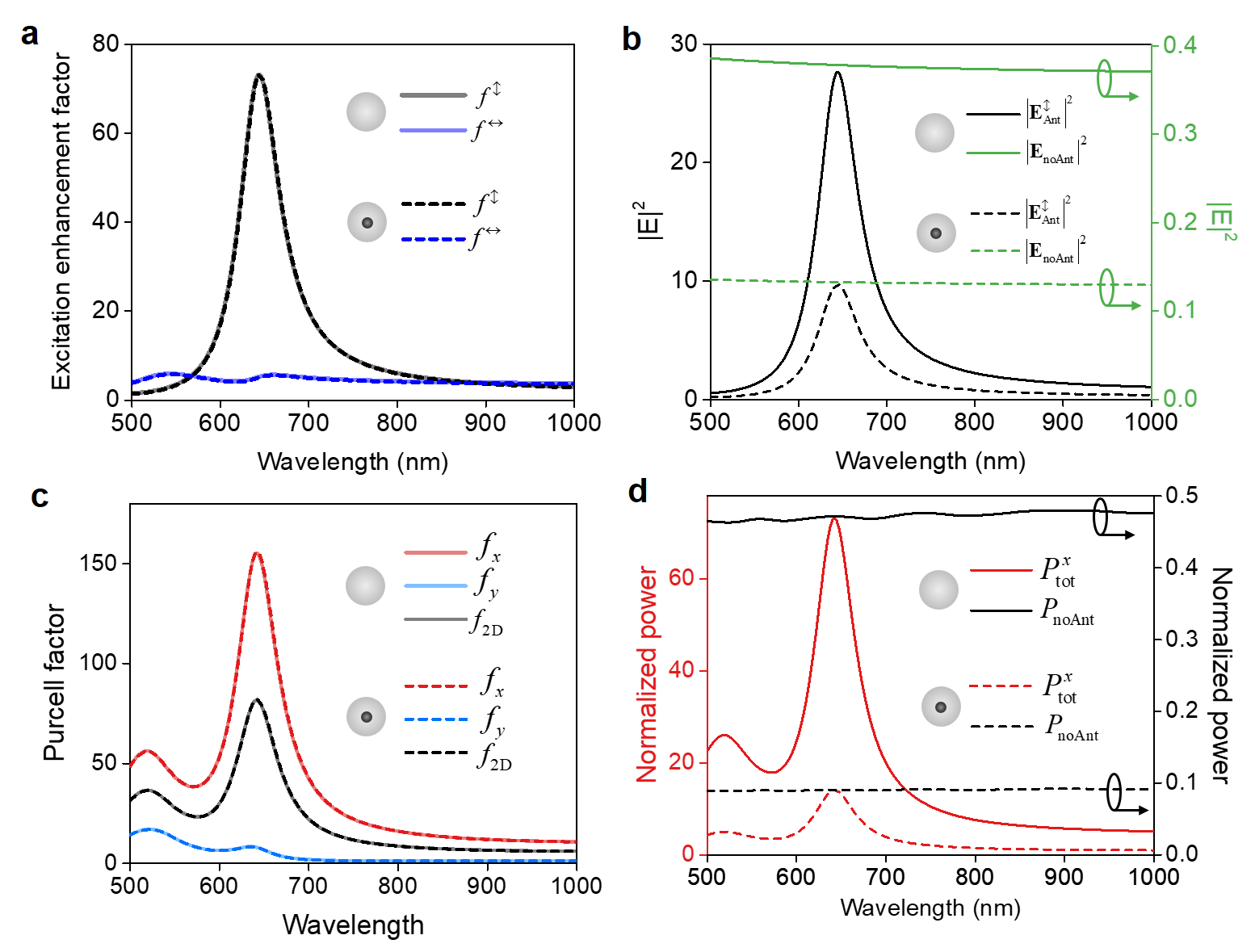


**Fig. S15** **The influence of the high refractive index of the QD itself on the simulated excitation enhancement factor and Purcell factor**. (**a**) Simulated excitation enhancement factor without (solid curves) and with (dashed curves) the QD itself. For the simulations, the diameter of the QD itself is set as 8 nm and the refractive index of the QD itself is set as 2.6. (**b**) Simulated local field normalized to the incident field. For clarity, only the result for the *y*-polarized excitation is shown in this panel. (**c**) Simulated Purcell factor without (solid curves) and with (dashed curves) the QD itself. (**d**) Simulated dipole power normalized to the vacuum dipole power *P*_0_. For clarity, only the result for the *x*-oriented dipole is shown in this panel.

## Section S15. Influence of the plane wave approximation on the numerical simulations

Plane wave excitation is a reasonable approximation of the Gaussian beam excitation for our experiment, where the Gaussian beam is loosely focused to a focal spot with a diameter of ~ 2μm (the numerical aperture of the objective is not fully used). To confirm this, we re-simulated the excitation enhancement spectra under *y*-polarized, *x*-polarized and elliptically polarized excitations using a Gaussian beam whose focal spot diameter is 2 μm and compared the results with those simulated using plane wave excitations. The comparison is shown in Fig. S16, which shows that the difference is within 5% for the whole spectral range.


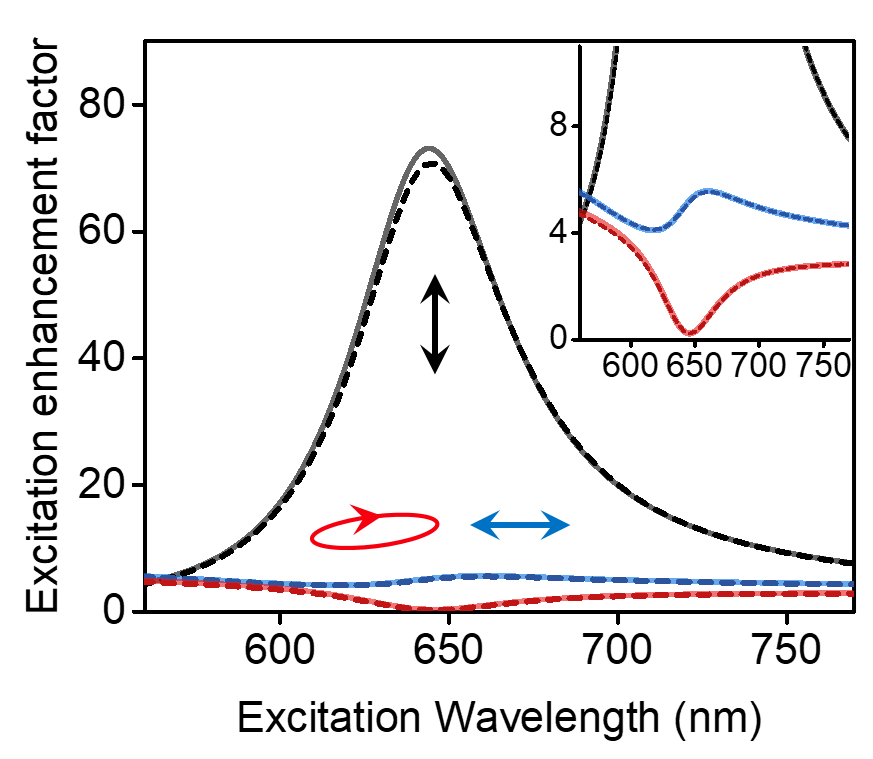


**Fig. S16 Comparison between plane wave excitation and Gaussian beam excitation.** The solid curves are the results simulated with plane wave excitation, the dashed lines are the results simulated with Gaussian beam excitation. The Gaussian beam has a focal spot diameter of ~ 2μm, as in the experiment.

## References for this Supplementary Information

1. Stockman, M. I., Faleev, S. V. & Bergman, D. J. Coherent control of femtosecond energy localization in nanosystems. *Physical Review Letters* **88**, 067402 (2002).

2. Aeschlimann, M., Bauer, M., Bayer, D., Brixner, T., Cunovic, S., Dimler, F., Fischer, A., Pfeiffer, W., Rohmer, M., Schneider, C., Steeb, F., Struber, C. & Voronine, D. V. Spatiotemporal control of nanooptical excitations. *Proceedings of the National Academy of Sciences of the United States of America* **107**, 5329-5333 (2010).

3. Brinks, D., Castro-Lopez, M., Hildner, R. & van Hulst, N. F. Plasmonic antennas as design elements for coherent ultrafast nanophotonics. *Proceedings of the National Academy of Sciences of the United States of America* **110**, 18386-18390 (2013).

4. Johnson, P. B. & Christy, R. W. Optical Constants of the Noble Metals. *Physical Review B* **6**, 4370-4379 (1972).

5. Vezzoli, S., Shojaii, S., Cialdi, S., Cipriani, D., Castelli, F., Paris, M. G. A., Carbone, L., Cozzoli, P. D., Giacobino, E. & Bramati, A. An ensemble-based method to assess the quality of a sample of nanocrystals as single photon emitters. *Optics Communications* **300**, 215-219 (2013).

6. Yao, J., Larson, D. R., Vishwasrao, H. D., Zipfel, W. R. & Webb, W. W. Blinking and nonradiant dark fraction of water-soluble quantum dots in aqueous solution. *Proceedings of the National Academy of Sciences of the United States of America* **102**, 14284-14289 (2005).

7. Chen, O., Zhao, J., Chauhan, V. P., Cui, J., Wong, C., Harris, D. K., Wei, H., Han, H. S., Fukumura, D., Jain, R. K. & Bawendi, M. G. Compact high-quality CdSe-CdS core-shell nanocrystals with narrow emission linewidths and suppressed blinking. *Nature Materials* **12**, 445-451 (2013).

8. Qin, H. Y., Meng, R. Y., Wang, N. & Peng, X. G. Photoluminescence Intermittency and Photo-Bleaching of Single Colloidal Quantum Dot. *Advanced Materials* **29**, 1606923 (2017).

9. Brokmann, X., Coolen, L., Dahan, M. & Hermier, J. P. Measurement of the radiative and nonradiative decay rates of single CdSe nanocrystals through a controlled modification of their spontaneous emission. *Physical Review Letters* **93**, 107403 (2004).

10. Fisher, B. R., Eisler, H. J., Stott, N. E. & Bawendi, M. G. Emission intensity dependence and single-exponential behavior in single colloidal quantum dot fluorescence lifetimes. *Journal of Physical Chemistry B* **108**, 143-148 (2004).

11. Spinicelli, P., Buil, S., Quélin, X., Mahler, B., Dubertret, B. & Hermier, J. P. Bright and Grey States in CdSe-CdS Nanocrystals Exhibiting Strongly Reduced Blinking. *Physical Review Letters* **102**, 136801 (2009).

12. Javaux, C., Mahler, B., Dubertret, B., Shabaev, A., Rodina, A. V., Efros, A. L., Yakovlev, D. R., Liu, F., Bayer, M., Camps, G., Biadala, L., Buil, S., Quelin, X. & Hermier, J. P. Thermal activation of non-radiative Auger recombination in charged colloidal nanocrystals. *Nature Nanotechnology* **8**, 206-212 (2013).

13. Xu, W. W., Hou, X. Q., Meng, Y. J., Meng, R. Y., Wang, Z. Y., Qin, H. Y., Peng, X. G. & Chen, X. W. Deciphering Charging Status, Absolute Quantum Efficiency, and Absorption Cross Section of Multicarrier States in Single Colloidal Quantum Dots. *Nano Letters* **17**, 7487-7493 (2017).

14. Empedocles, S. A., Neuhauser, R. & Bawendi, M. G. Three-dimensional orientation measurements of symmetric single chromophores using polarization microscopy. *Nature* **399**, 126-130 (1999).

15. Krauss, T. D., O'Brien, S. & Brus, L. E. Charge and photoionization properties of single semiconductor nanocrystals. *Journal of Physical Chemistry B* **105**, 1725-1733 (2001).
